# Dynamic Metabolic States in TNBC: Orchestrating Spatiotemporal Adaptation and Therapy

**DOI:** 10.32604/or.2026.085967

**Published:** 2026-08-13

**Authors:** Yida Wang, Haiyue You, Jingyi Gao, Feng Zhang, Xin Ning, Xinfeng Yang, Zhiwen Qian, Ying Jiang, Lu Liu, Danping Wu, Yanfang Gu, Daozhen Chen, Yan Zhang

**Affiliations:** 1 Department of Oncology, Wuxi Maternal and Child Health Care Hospital, Wuxi Medical Center, Nanjing Medical University, Wuxi, China; 2 Department of Oncology, Wuxi Maternity and Child Health Care Hospital, Women’s Hospital of Jiangnan University, Jiangnan University, Wuxi, China; 3 Suzhou Medical College, Soochow University, Suzhou, China

**Keywords:** Triple-negative breast cancer, metabolic plasticity, flux valves, tumor microenvironment, spatial omics

## Abstract

Triple-negative breast cancer (TNBC) is characterized by marked metabolic plasticity, spatial heterogeneity, and therapy-induced adaptive remodeling. However, TNBC metabolism is often discussed as isolated pathways, making it difficult to link metabolic rewiring to immune exclusion, drug-tolerant persister cells, and treatment windows. Here, we propose a functional metabolic operating-state framework to organize recurrent adaptive programs in TNBC. Importantly, the S1–S5 framework is not a clinically validated subtype classification, but a set of coexisting and reversible operating states shaped by microenvironmental and therapeutic pressures. S1 represents a glycolysis–lactate/acidosis barrier; S2 denotes fatty acid oxidation (FAO)/oxidative phosphorylation (OXPHOS)-supported persister-like survival; S3 reflects NADPH and one-carbon metabolism-mediated reductive defense; S4 captures lipogenesis, cholesterol metabolism, and membrane remodeling; and S5 represents a ferroptosis tipping window emerging when redox and lipid-peroxide defenses fail. We further highlight flux-valve nodes that redirect carbon, nitrogen, lipid, and redox allocation, thereby biasing transitions among these states. Integrating evidence from spatial omics, metabolic imaging, tumor immunology, and therapeutic studies, we discuss how metabolic isozones reinforce immune suppression and how staged interventions may exploit state-specific vulnerabilities. This framework is intended to generate monitorable and falsifiable decision hypotheses for biomarker development, combination therapy design, and future prospective validation in TNBC.

## 1 Introduction

Breast cancer is among the most common malignancies in women worldwide. Triple-negative breast cancer (TNBC) accounts for approximately 15% to 20% of all breast cancers and is defined by the lack of estrogen receptor, progesterone receptor, and human epidermal growth factor receptor 2 (HER2) expression; clinically, it is characterized by high invasiveness, an elevated risk of metastasis, and poor prognosis [1]. At present, management of TNBC still relies primarily on surgery, adjuvant chemotherapy, and radiotherapy, yet a substantial fraction of patients have already missed the opportunity for curative surgery at diagnosis [2], placing greater demands on systemic treatment strategies and on the ability to predict therapeutic benefit.

In recent years, metabolism-targeted therapy has emerged as a major focus in TNBC research. Metabolic reprogramming not only supports rapid proliferation and metastatic dissemination, but also participates in shaping responses to chemotherapy and targeted agents [3]. Despite steady progress, however, current understanding of TNBC metabolic features often remains confined to individual pathways or molecules, and a conceptual framework that integrates diverse adaptive programs from the perspective of functional operation and links them to therapeutic response remains lacking.

Tumor cells exhibit marked metabolic adaptability: under adverse conditions within the tumor microenvironment (TME), including hypoxia, nutrient scarcity, acid-base imbalance, and oxidative stress, they can flexibly switch between metabolic modes through reprogramming, thereby effectively recalibrating cellular bioenergetic and biosynthetic processes [4,5,6]. These states are not isolated; rather, they display continuous variation and reversible interconversion driven by oxygen tension, nutrient availability, and stress cues [e.g., reactive oxygen species (ROS) and chemotherapy pressure], forming a function-oriented set of metabolic operating states.

**Figure 1:**
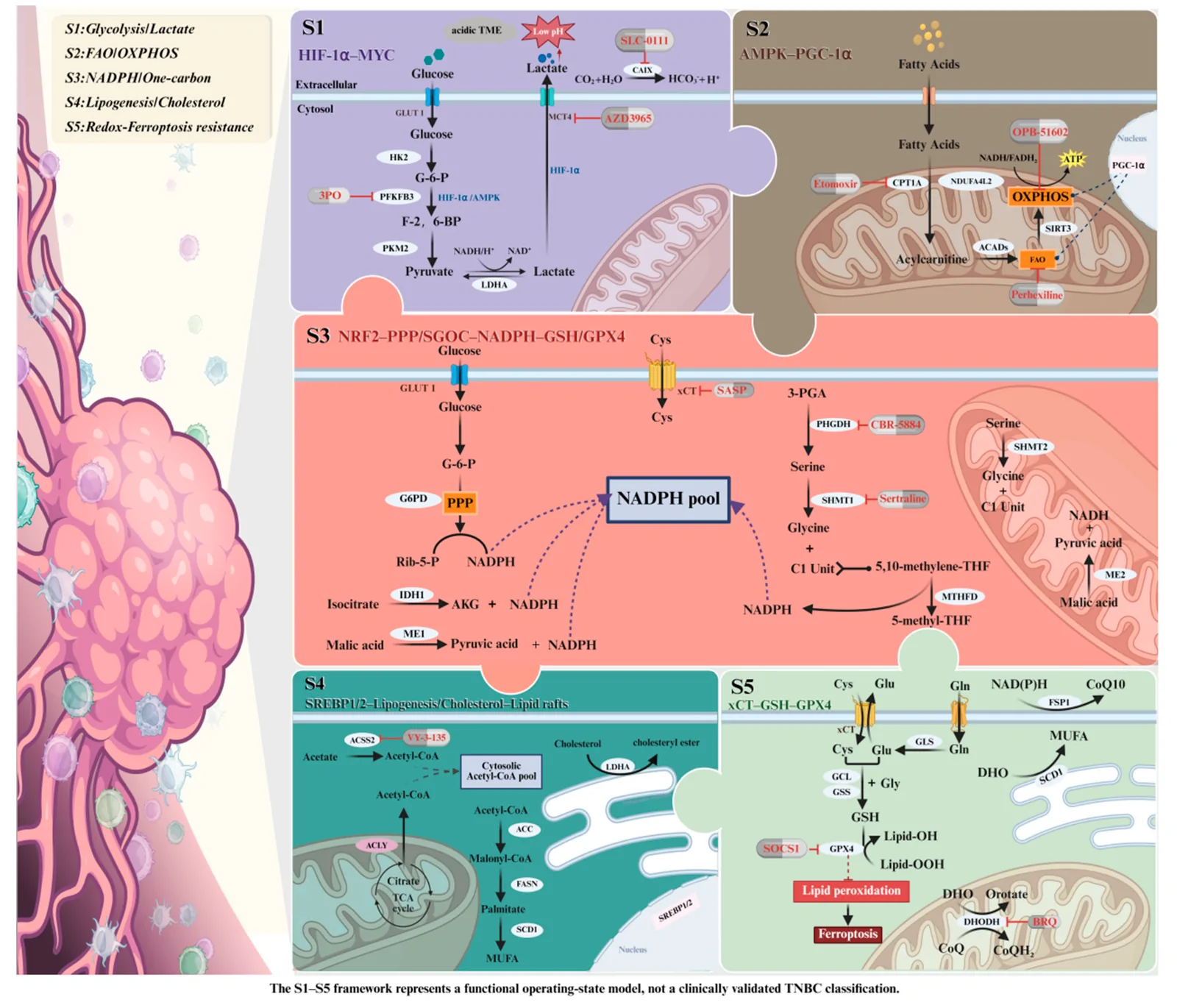
**Functional S1–S5 metabolic operating-state spectrum in triple-negative breast cancer (TNBC)**. This schematic summarizes five recurrent and reversible metabolic operating states shaped by microenvironmental and therapeutic pressures: S1, glycolysis-lactate/acidity; S2, fatty acid oxidation (FAO)/oxidative phosphorylation (OXPHOS)-supported persistence; S3, NADPH/one-carbon/glutathione (GSH)-mediated reductive defense; S4, lipogenesis/cholesterol-driven membrane remodeling; and S5, ferroptosis vulnerability when redox and lipid-peroxide defenses fail. The interlocking design emphasizes coexistence, reversibility, and transition tendencies, rather than a clinically validated TNBC subtype classification. Created in BioRender.com.

Accumulating evidence indicates that, against the backdrop of metabolic plasticity in TNBC, recurrent microenvironmental and therapeutic selection pressures often drive tumor cells to converge on a limited number of metabolic operating modes with clear functional priorities [4,7]. Accordingly, we use the concept of metabolic operating states to interpret this plasticity and summarize five commonly recurring states (S1–S5), which reflect distinct metabolic orientations in energy acquisition, redox homeostasis, lipid balance, and cell-fate decision-making [7]. Importantly, the S1–S5 states are not clinically validated TNBC classifications, but functional metabolic operating states that describe dynamic flux redistribution under microenvironmental and therapeutic pressures. The central conclusion of this review is that TNBC metabolic plasticity, although complex, repeatedly converges on several functional priorities, including lactate–acid barrier formation, FAO/OXPHOS-supported persister-like survival, NADPH/one-carbon-mediated reductive defense, lipid remodeling, and ferroptosis-related vulnerability. We therefore discuss the functional features, key regulatory nodes, state-switching logic, and therapeutic implications of this framework as a testable operating map for understanding TNBC metabolic plasticity and guiding future biomarker-driven translational research (Fig. 1).

## 2 Metabolic State Spectrum and Flux Switches in TNBC

### 2.1 State S1: The Glucose-Lactate Program (Warburg Metabolism and Lactate Export)

#### 2.1.1 Definition and Activation Cues

Here, we define the S1 state as a metabolic operating mode whose primary objective is rapid energy acquisition coupled to lactate export. In this state, TNBC cells prioritize glucose uptake and glycolytic flux, and even in the presence of oxygen, they actively restrain mitochondrial pyruvate oxidation and instead route carbon toward lactate production and extrusion. This program is governed by the hypoxia-inducible factor-1α (HIF-1α)-MYC axis: under hypoxia (<2% O_2_), stabilized HIF-1α cooperates with c-MYC to transcriptionally activate glucose transporter 1 (GLUT1), hexokinase 2 (HK2), 6-phosphofructo-2-kinase/fructose-2,6-bisphosphatase 3 (PFKFB3), lactate dehydrogenase A (LDHA), and monocarboxylate transporter 4 (MCT4), thereby boosting glucose uptake, accelerating glycolysis, and promoting lactate generation and export, establishing a metabolic state dominated by high-throughput glycolysis-driven lactate production. When glucose becomes limiting or GLUT1/HK2 is inhibited, cells are forced to switch into alternative states [8]. To sustain high flux, PFKFB3 is phosphorylated and activated, producing abundant fructose-2,6-bisphosphate (F-2,6-BP), which promotes pyruvate dehydrogenase kinase 1 (PDK1) activation and pyruvate dehydrogenase (PDH) inhibition, blocks pyruvate entry into the tricarboxylic acid (TCA) cycle, and enforces carbon diversion to lactate while suppressing conversion of S1 into other states [9,10]. This culminates in an immunosuppressive acidic microenvironment, i.e., the classic Warburg effect [11,12]. Although S1 rapidly satisfies the energetic and biosynthetic demands of proliferating tumor cells, it does so at the cost of suppressed mitochondrial metabolism, reduced metabolic efficiency, and a strict dependence on continuous glucose supply, rendering the state readily switchable and therapeutically vulnerable under nutrient restriction or flux blockade [13].

#### 2.1.2 Key Nodes and Biological Functions

Key nodes in the S1 state include GLUT1 (SLC2A1), which mediates hypoxia-driven glucose uptake [13]; HK2, which supports glycolytic entry and mitochondrial apoptosis resistance [14]; PFKFB3, which functions as a glycolytic accelerator downstream of HIF-1α and AMPK-related signaling [9]; PKM2, which balances lactate production with diversion of glycolytic intermediates into biosynthesis and can also act as a nuclear co-regulator [11,15,16]; LDHA, which regenerates NAD^+^ and sustains glycolytic flux [17,18]; MCT4/SLC16A3, which exports lactate and prevents intracellular acidification [19]; and CAIX, which maintains intracellular pH while contributing to extracellular acidification [20].

Functionally, S1 provides rapid ATP production despite low per-glucose efficiency [21], supplies glycolytic intermediates for nucleotide, amino acid, and lipid biosynthesis [22], and constructs an acidic immunosuppressive microenvironment. Lactate accumulation and reduced extracellular pH suppress CD8^+^ T-cell proliferation, cytotoxicity, and IFN-γ secretion, while promoting regulatory T cells, myeloid-derived suppressor cells, and programmed death-ligand 1 (PD-L1) expression through HIF-1α- and NFAT-related mechanisms [18]. Lactate also promotes epithelial-to-mesenchymal transition (EMT), migration, and invasion through transforming growth factor β (TGF-β), NF-κB, and Notch signaling [23]. Consistently, high expression of S1-associated nodes, including PFKFB3, MCT4, LDHA, and CAIX, has been associated with chemotherapy resistance, early relapse, and poor prognosis in TNBC [20,24].

#### 2.1.3 Therapeutic Vulnerabilities and Adaptive Escape

Therapeutically, S1 represents a lactate–acidity barrier that can be disrupted by targeting glycolytic flux, lactate export, or extracellular acidosis. MCT1/4 inhibition can induce intracellular acid stress and apoptosis [23,24], whereas AZD3965 has shown preclinical activity but limited monotherapy efficacy in early-phase trials, potentially because acid stress activates AMPK-dependent autophagy and promotes a switch toward the mitochondria-dependent S2 state [24]. Similarly, the PFKFB3 inhibitor 3PO reduces glycolysis, proliferation, and chemoresistance in preclinical TNBC models [25], while the CAIX inhibitor SLC-0111 may alleviate acidosis, improve immune infiltration, and synergize with immune checkpoint blockade in preclinical settings [26]. Therefore, S1 blockade is unlikely to be sufficient as monotherapy and should be coupled with strategies that prevent adaptive state switching, such as combining MCT or glycolytic inhibition with autophagy blockade, LDHA inhibition, or immune checkpoint blockade [27,28,29]. This AMPK-mediated transition toward S2-like mitochondrial dependence may partly explain why glycolysis inhibitors often show limited clinical activity as single agents [24,29].

### 2.2 State S2: FAO/OXPHOS (Mitochondrial, Persister-Like)

#### 2.2.1 Definition and Switching Cues

Here we define the S2 state as a metabolic operating mode whose primary objective is long-term energy homeostasis and survival sustained by mitochondrial oxidative metabolism. In this state, TNBC cells redirect flux away from glucose-centered rapid glycolysis toward FAO and oxidative phosphorylation (OXPHOS), thereby achieving greater energetic efficiency and reducing dependence on exogenous glucose [30,31]. S2 is typically engaged under glucose limitation, lactate accumulation, acidic stress, or therapy-induced pressure, including chemotherapy and targeted therapy [32]. Functionally, S2 supports a low-proliferative but highly viable persister-like phenotype, allowing TNBC cells to survive adverse microenvironmental and therapeutic conditions [31,32]. While S2 markedly enhances survival under adverse conditions, it also depends on intact mitochondria and sustained oxidative metabolism, creating specific vulnerabilities to mitochondrial perturbation and blockade of fatty acid flux [30,32].

#### 2.2.2 FAO/OXPHOS Nodes and Persister-Like Survival

The S2 program is driven primarily by the AMP-activated protein kinase (AMPK)–peroxisome proliferator-activated receptor gamma coactivator 1-alpha (PGC-1α) axis. Upon glucose restriction or chemotherapy challenge, the energy sensor AMPK detects an increased AMP/ATP ratio and activates downstream PGC-1α. Concurrently, carnitine palmitoyltransferase 1 (CPT1A) expression is upregulated, opening the “flux gate” for long-chain fatty acids to enter mitochondria and promoting a switch from S1 to S2 [33,34,35]. In S2, tumor cells preferentially rely on FAO and OXPHOS to maintain ATP production and survival; long-term persistence requires functional mitochondria to sustain energy supply and cellular homeostasis [36]. Consistently, FAO inhibition can disrupt this persister-like state, forcing metabolic regression or cell death under conditions where mitochondrial metabolism becomes indispensable [37,38].

Key S2 nodes include CPT1A and acyl-CoA dehydrogenases (ACADs), which control fatty acid mitochondrial entry and β-oxidation [37,38]. In TNBC, CPT1A and related FAO enzymes are associated with enhanced OXPHOS, survival, and drug resistance [30,39,40], whereas ACAD-dependent FAO contributes to redox balance and stress adaptation [38]. PGC-1α functions as a central transcriptional co-activator of mitochondrial biogenesis, FAO, and OXPHOS [34], and has been linked to TNBC metastatic capacity [35]. NDUFA4L2 may further reshape respiratory complex I activity under hypoxia [41], limiting excessive reactive oxygen species (ROS) and supporting survival in oxidative environments [42]. In addition, sirtuin 3 (SIRT3) and nicotinamide phosphoribosyltransferase (NAMPT) help maintain mitochondrial function and NAD^+^-dependent stress adaptation [43]; NAMPT-mediated NAD^+^ biosynthesis may support SIRT3 activity, suppress glucose-deprivation-induced oxidative stress, and promote metabolic reprogramming in TNBC [44].

The biological advantage of S2 lies in its ability to trade proliferative speed for metabolic durability. By relying on FAO/OXPHOS, S2 cells can maintain ATP production in nutrient-poor, acidic, or post-treatment niches, where glucose-centered glycolysis becomes less stable [45,46]. This mitochondrial state also helps buffer oxidative stress: cancer stem-like cells may use FAO-derived reducing equivalents together with antioxidant systems such as Prx3, SOD2, and GPX4 to sustain mitochondrial energy production while avoiding excessive ROS-mediated damage [45,47]. Under EGFR-TKI, BRAF inhibitor, or chemotherapy pressure, resistant tumor cells often converge on this survival pattern, characterized by enhanced OXPHOS and FAO, reduced glycolytic dependence, autophagy activation, anti-apoptotic buffering, and a dormancy-persister phenotype that allows transient escape from drug killing [32,48].

#### 2.2.3 Therapeutic Opportunities and Toxicity Concerns

These features make S2 less suitable for immediate cytoreduction but highly relevant to post-treatment residual disease. Complex I inhibitors such as IACS-010759 and OPB-51602 can suppress mitochondrial OXPHOS [49], and early-phase clinical data suggest that tumors with strong mitochondrial dependence may be particularly vulnerable to this strategy [50]. In paclitaxel-treated residual TNBC cells, increased CPT1B expression and FAO flux have been linked to acquired chemotherapy resistance [39,40]. Accordingly, the CPT1 inhibitor etomoxir can reduce FAO, ATP production, proliferation, and metastatic traits [51], while perhexiline can enhance chemosensitivity and strengthen paclitaxel activity in resistant TNBC models [52]. OXPHOS-active TNBC cells may also become more dependent on anti-apoptotic BCL-XL, although the activity of BCL-XL inhibitors such as navitoclax, A1155463, and A1331852 may be modified by cancer-associated fibroblasts and other microenvironmental factors [32,51]. Because FAO/OXPHOS and complex I activity are also essential in normal high-energy tissues, particularly the heart and nervous system, S2-directed interventions require cautious dose scheduling, toxicity monitoring, and biomarker-guided patient selection. Thus, the most plausible use of S2-targeted therapy is not frontline monotherapy, but post-treatment maintenance or persister eradication after cytotoxic or immune-based therapy [32,50].

### 2.3 State S3: NADPH and One-Carbon-Coupled Antioxidant Defense

#### 2.3.1 Reductive-Defense State and Activation Cues

Here, we define the S3 state as a metabolic operating mode centered on redox preservation rather than maximal energy production or rapid proliferation. Under radiotherapy, chemotherapy, inflammatory stress, or lipid-peroxidation pressure, TNBC cells can reallocate carbon flux and reducing power toward NADPH regeneration, glutathione recycling, and one-carbon-supported antioxidant defense [53]. In this context, NADPH becomes a limiting resource that is preferentially regenerated and conserved to delay oxidative injury, maintain GPX4 activity, suppress lipid peroxidation, and prevent ferroptotic collapse [54]. Thus, S3 represents a reductive-defense state that allows tumor cells to survive transient but intense oxidative stress.

The establishment of S3 is largely coordinated by nuclear factor erythroid 2-related factor 2 (NRF2)-driven metabolic reprogramming. When treatment-induced reactive oxygen species (ROS) accumulate, NRF2 activates genes involved in NADPH production, glutathione synthesis, cystine uptake, and one-carbon metabolism, thereby shifting flux away from simple glycolytic or mitochondrial output toward reducing-power supply [55,56,57]. Multiple NADPH-generating branches contribute to this state, including the pentose phosphate pathway (PPP), malic enzyme 1/2 (ME1/ME2), and isocitrate dehydrogenase 1/2 (IDH1/IDH2), which replenish reducing equivalents through glucose-6-phosphate, malate, and isocitrate-centered reactions [58,59]. In parallel, the serine–glycine–one-carbon (SGOC) pathway functions as a coupling amplifier: the phosphoglycerate dehydrogenase (PHGDH)–serine hydroxymethyltransferase (SHMT)–MTHFD axis channels serine-derived carbon into folate-dependent one-carbon metabolism, supporting nucleotide synthesis, methyl-donor generation, and additional NADPH production [60,61]. Therefore, in S3, one-carbon metabolism is not only an anabolic pathway but also a functional component of the reductive-defense network [62].

#### 2.3.2 NADPH, One-Carbon, and GSH-Related Nodes

Key S3 nodes include G6PD, the rate-limiting enzyme of the PPP, which generates NADPH while supporting ribose-5-phosphate production for nucleotide synthesis [63]. In TNBC, G6PD contributes to antioxidant capacity, anabolic growth, cytokine regulation, immune remodeling, migration, and metastasis, making it a candidate therapeutic node [64]. ME1/ME2 and IDH1/IDH2 further support NADPH-dependent redox buffering and metabolic adaptation [65,66,67]. SGOC enzymes, including PHGDH, SHMT1/2, and MTHFD family members, connect serine metabolism with nucleotide synthesis, methylation reactions, and redox homeostasis [68]; PHGDH is frequently overexpressed in TNBC and has been associated with invasiveness, metastasis, and poor prognosis [69]. The xCT/GPX4 axis represents another central S3 barrier: SLC7A11/xCT imports cystine to support glutathione synthesis [70], whereas GPX4 uses glutathione to detoxify lipid peroxides and preserve membrane integrity [71]. High SLC7A11 and GPX4 expression can therefore promote TNBC survival while creating exploitable dependencies for ferroptosis-oriented intervention [72,73,74].

Biologically, S3 protects TNBC cells from oxidative and lipid-peroxidation stress through three linked functions. First, PPP, ME/IDH, and SGOC pathways regenerate NADPH to sustain glutathione reductase, thioredoxin reductase, and other antioxidant systems [75]. Second, SGOC metabolism supplies one-carbon units for nucleotide synthesis and methylation reactions, allowing stressed tumor cells to maintain repair and proliferative capacity [76]. Third, NADPH and glutathione availability preserve GPX4 activity, restrict lipid peroxide accumulation, and reduce ferroptosis sensitivity [77]. These redox-buffering programs may also reshape the immune microenvironment by lowering oxidative pressure and favoring regulatory T cells and myeloid-derived suppressor cells, thereby contributing to immune evasion [78,79].

#### 2.3.3 Timed Therapeutic Disruption of Redox Buffering

Therapeutically, S3 is best viewed as a short-window vulnerability created by oxidative therapy rather than a state suited for prolonged nonspecific suppression. SHMT inhibition, xCT blockade, PHGDH targeting, MTHFD2 inhibition, ME2 suppression, and NAMPT inhibition may all weaken NADPH or glutathione-dependent defenses, but these pathways are also important in normal proliferating and immune cells. The SHMT inhibitor sertraline can reduce one-carbon flux, alter methylation and antioxidant capacity, and resensitize resistant TNBC cells to doxorubicin [80]; when combined during the oxidative window induced by radiotherapy or platinum chemotherapy, it may intensify ROS accumulation [81]. The xCT inhibitor sulfasalazine (SASP) blocks cystine/glutamate exchange, reduces glutathione synthesis, and suppresses tumor proliferation, supporting xCT as a candidate TNBC target [82,83,84]. Additional approaches include ME2 inhibition [85], combined MTHFD2 and checkpoint kinase inhibition [86], NAMPT inhibition to perturb NAD^+^/NADPH-related metabolic adaptation [87], and PHGDH inhibition with agents such as CBR-5884 in PHGDH-high TNBC [88]. However, because NADPH regeneration, one-carbon metabolism, and glutathione-dependent redox buffering are not tumor-exclusive, S3-directed strategies require biomarker selection, intermittent scheduling, and careful monitoring for myelosuppression, immune-cell dysfunction, and systemic metabolic toxicity. Therefore, the most rational use of S3 targeting is a timed reductive-defense breach during radiotherapy, platinum therapy, or other ROS-inducing treatments, rather than long-term unselected antioxidant-pathway blockade.

### 2.4 State S4: Lipogenesis, Membrane Remodeling, and Signaling Lipid Rafts

#### 2.4.1 Lipid-Remodeling State and Spatial Enrichment

Here, we define the S4 state as a metabolic operating mode centered on membrane remodeling and lipid-signaling architecture. In this state, TNBC cells do not merely accumulate lipids for storage or energy supply; instead, they actively remodel fatty acid, phospholipid, and cholesterol composition to optimize receptor signaling, adhesion, migration, and adaptation to organ-specific niches [89,90]. S4 is largely coordinated by sterol regulatory element-binding protein 1/2 (SREBP1/2)-dependent lipogenic programs, which are closely linked to tumor initiation, invasion, and metastasis [91]. This state is frequently enriched at invasive fronts and in metastatic contexts, including brain metastasis, where acetyl-CoA-generating enzymes such as ATP-citrate lyase (ACLY), acetyl-CoA synthetase 2 (ACSS2), and fatty acid synthase (FASN) support fatty acid and cholesterol synthesis [92]. Under hypoxia or nutrient deprivation, when glucose-derived acetyl-CoA becomes limited, ACSS2-mediated acetate recycling provides an alternative acetyl-CoA source to sustain lipogenesis, survival, and proliferation [93]. In parallel, increased cholesterol synthesis and cholesterol metabolite accumulation can reshape the tumor microenvironment, enhance acidity and oxidative stress, suppress CD8^+^ T-cell function, and reduce immune checkpoint blockade (ICB) responsiveness [94]. Thus, S4 enhances invasion and microenvironmental adaptation, but its dependence on lipid flux and membrane homeostasis also creates vulnerabilities to lipid-metabolic and membrane-disruptive interventions [95,96].

#### 2.4.2 Lipogenic and Cholesterol-Related Nodes

The core machinery of S4 includes multiple acetyl-CoA, fatty acid, and cholesterol-processing nodes. ACLY generates cytosolic acetyl-CoA from citrate and provides a key substrate for fatty acid and cholesterol synthesis [97]; it is frequently overexpressed or hyperactivated in TNBC [98,99]. ACSS2 converts acetate into acetyl-CoA and becomes particularly important under nutrient stress or hypoxia [100]. Acetyl-CoA carboxylase (ACC) produces malonyl-CoA, a rate-limiting substrate for fatty acid synthesis [101], whereas FASN catalyzes *de novo* fatty acid production and has been implicated in TNBC progression and breast cancer brain metastasis [102]. Stearoyl-CoA desaturase 1 (SCD1) regulates monounsaturated fatty acid production and can influence ferroptosis sensitivity; for example, salidroside may sensitize TNBC to ferroptosis by suppressing SCD1-mediated lipid remodeling [103]. Sterol O-acyltransferase 1 (SOAT1/ACAT1) converts free cholesterol into cholesteryl esters and represents another cholesterol-homeostasis node with antitumor potential. Upstream, SREBP1/2 integrate lipid synthesis and uptake programs [104], and SREBP-1 mRNA regulation has been linked to TNBC proliferation, invasion, and migration [92,105].

What distinguishes S4 from general lipogenesis is its impact on membrane organization and signaling. ACSL4-mediated incorporation of polyunsaturated fatty acids into phospholipids can promote integrin β1-dependent CD47 lipid-raft localization and activation, thereby enhancing TNBC metastasis [106]. Cholesterol- and sphingolipid-enriched lipid rafts organize receptor complexes and protein-protein interactions, and cholesterol availability directly shapes raft dynamics and function [107]. Relatedly, StarD4 has been suggested to promote TNBC progression through cholesterol-pathway crosstalk and stabilization of ITGA5 protein [108]. S4 may also contribute to brain metastatic adaptation, a clinically important feature of TNBC [109,110]; reduced RARRES2 expression has been reported to support breast cancer cell survival in the brain microenvironment through the PTEN-mTOR-SREBP1 axis [111].

#### 2.4.3 Targeting Membrane Remodeling and Adaptive Bypass

Therapeutically, S4 is attractive because lipid remodeling links tumor invasion, immune escape, and drug delivery barriers. Targeting ACSL4 with PRGL493 can reduce unsaturated phospholipids and, when combined with paclitaxel and cisplatin, suppress TNBC growth and metastasis [106]. The ACSS2 inhibitor VY-3-135 blocks acetate-dependent acetyl-CoA production and may restrain TNBC progression while potentially preserving acetate availability for T-cell effector metabolism [112]. SREBP1-targeting approaches, including therapeutic proteins that promote SREBP1 mRNA degradation, can reduce lipid abundance and suppress TNBC proliferation and metastasis [92]. However, FASN inhibition alone may have limited efficacy because tumor cells can bypass *de novo* lipogenesis by importing exogenous lipids through CD36-mediated uptake [113,114]. This adaptive lipid compensation is also relevant to immune therapy: inhibition of B7-H3 may upregulate SREBP1 and FASN expression, suggesting a dynamic “immune attack–metabolic compensation” loop [115]. In addition, combining FASN inhibition with antibody–drug conjugates (ADCs) may disrupt lipid-raft architecture, enhance ADC endocytosis, and overcome ADC resistance, supporting a physical–metabolic combination strategy [116]. Overall, S4-targeted therapy should be viewed not only as lipid synthesis blockade, but also as an approach to weaken membrane signaling, metastatic fitness, immune escape, and drug-delivery resistance.

### 2.5 State S5: Redox Collapse and the Ferroptotic Tipping Point

#### 2.5.1 Ferroptotic Tipping Point and Redox-Defense Failure

We define the S5 state as a fate-critical metabolic boundary marked by uncontrolled lipid peroxidation and ferroptotic commitment. Unlike S1–S4, which represent adaptive operating states for metabolic maintenance, S5 is not a stable functional state. Rather, it emerges when the redox-buffering capacity of TNBC cells can no longer counteract iron-dependent reactive oxygen species (ROS)-driven peroxidation of polyunsaturated fatty acid (PUFA)-containing membrane lipids [117,118]. This transition is mainly precipitated by failure of the glutathione (GSH)/glutathione peroxidase 4 (GPX4) and nicotinamide adenine dinucleotide phosphate (NADPH) defense axes [119]. Thus, S5 marks the shift from reversible metabolic adaptation to irreversible oxidative membrane damage, making it a potentially selective therapeutic window [120].

Conceptually, S5 is directly opposed to the S3 reductive-defense state. S3 raises the threshold for lipid peroxidation through the pentose phosphate pathway, one-carbon metabolism, NADPH regeneration, GSH synthesis, and GPX4 activity. When NADPH supply becomes insufficient, GSH is depleted, or GPX4 is inhibited, lipid peroxides can no longer be cleared efficiently and cells are pushed toward S5 [121,122]. Therefore, S5 is usually not initiated by a single pathway defect, but by the combined burden of therapy-induced ROS, altered membrane lipid composition, iron availability, and exhaustion of antioxidant compensation [118,119].

#### 2.5.2 xCT/GSH/GPX4 and Compensatory Ferroptosis Defenses

The core biochemical axis of S5 is the xCT/GSH/GPX4 lipid-peroxide defense system [123]. System xCT imports cystine, which is reduced to cysteine for GSH synthesis in an NADPH-dependent manner [124]. GSH then supports GPX4-mediated detoxification of lipid hydroperoxides, thereby preserving membrane integrity and suppressing ferroptosis [125]. When this axis fails, iron-dependent ROS and lipid peroxide accumulation drive membrane damage and ferroptotic death [126]. Membrane PUFA content further determines ferroptosis sensitivity [127]. For example, ACSL4 can incorporate PUFA flux into phospholipids, increasing ferroptosis susceptibility while also contributing to membrane plasticity and metastatic behavior [106]. Conversely, stearoyl-CoA desaturase 1 (SCD1)-mediated monounsaturated fatty acid production can reduce ferroptosis sensitivity and has been associated with poor outcomes in TNBC [103].

Several compensatory ferroptosis-suppressive nodes shape the S5 threshold. xCT supports cystine import and GSH synthesis [124], whereas glutaminase (GLS) and glutamate dehydrogenase (GLUD) influence glutamate metabolism and glutamine dependence in TNBC. Ferroptosis suppressor protein 1 (FSP1) protects cells through a GSH-independent FSP1–CoQ10–NAD(P)H axis. Dihydroorotate dehydrogenase (DHODH) has also been linked to ferroptosis resistance and TNBC progression; high DHODH expression is associated with poor prognosis, and DHODH inhibition may disrupt macropinocytosis-related survival programs [128]. Together, these systems form parallel antioxidant and membrane-protective barriers that determine whether oxidative stress remains buffered or crosses the S5 tipping point.

The biological significance of S5 lies in its vulnerability rather than its stability. TNBC cells with high oxidative load must maintain GSH, GPX4, NADPH, and auxiliary ferroptosis suppressors to prevent lipid-peroxide accumulation [124]. In ferroptosis-prone contexts, such as luminal androgen receptor (LAR)-like TNBC with increased PUFA/lipid peroxide features and GPX4 dependence, ferroptosis induction may create a therapeutic opportunity, particularly when combined with immune checkpoint blockade [129,130,131]. Consistently, high expression of Xc^−^/GSH/GPX4 pathway components and DHODH has been associated with early relapse and poor prognosis in TNBC [132,133].

#### 2.5.3 Ferroptosis-Oriented Therapeutic Windows

Therapeutically, S5 targeting aims to push tumor cells across the ferroptotic boundary rather than merely suppress proliferation. Agents that inhibit the xCT/GSH/GPX4 axis can deplete antioxidant capacity, accelerate lipid-peroxide accumulation, and enhance antitumor efficacy in TNBC and other cancers [125]. For example, progesterone receptor membrane component 1 (PGRMC1) may increase ferroptosis-mediated cell death by suppressing GPX4 in TNBC [131], whereas SOCS1 has been implicated in TNBC ferroptosis through GPX4-related regulation [132]. DMOCPTL, a parthenolide derivative, can bind GPX4, promote its ubiquitination, induce ferroptosis, and inhibit TNBC cell growth [133]. In parallel, the DHODH inhibitor brequinar (BRQ) can suppress macropinocytosis-associated survival in TNBC, and BRQ combined with anti-PD-1 therapy may overcome anti-PD-1 resistance and more effectively inhibit TNBC progression [128]. Overall, S5-directed therapy is most rational when timed to coincide with oxidative pressure, lipid-peroxidation priming, or failure of S3-like antioxidant compensation, rather than used as untargeted long-term redox disruption (Table 1).

A major translational constraint for metabolism-targeted therapy is therapeutic selectivity. Many candidate targets discussed in this review, including complex I, NAMPT, DHODH, one-carbon metabolism enzymes, and xCT/GPX4-related redox-buffering systems, also support essential metabolic functions in normal tissues. Therefore, systemic inhibition may produce narrow therapeutic windows and clinically relevant toxicities, including cardiac or neurologic toxicity, myelosuppression, gastrointestinal intolerance, hepatic metabolic disturbance, or impaired immune-cell function. In this context, the proposed short-course induction, barrier-breaking, or maintenance-blockade strategies should be interpreted as biomarker-guided and safety-constrained hypotheses rather than immediately applicable clinical schedules.

**Table 1:** Key metabolic nodes in TNBC: functions, evidence, agents, and potential toxicities with mitigation strategies.

Module	Key Node/Flux Valve	Primary Function/Phenotype	Investigational/Approved Agents	Evidence Stage/Phase and Disease Context	Trial ID	Potential Toxicity/Translational Limitation	Potential Mitigation or Monitoring Considerations
Glycolysis–lactate (S1)	PFKFB3	Increases fructose-2,6-bisphosphate to boost glycolysis	PFK-158	Phase I in advanced solid tumors; TNBC evidence remains preclinical	NCT02044861	Clinical safety and efficacy remain incompletely defined; adaptive diversion to the PPP, glutamine metabolism, or OXPHOS may limit activity	Consider short-course dosing, glucose/metabolic monitoring, and combination only with mechanistically justified blockade of bypass routes
	PKM2	Promotes glycolytic shunting and nuclear functions	TP-1454	Phase I in advanced metastatic or progressive solid tumors and anal cancer; not TNBC-specific	NCT04328740	The consequences of PKM2 activation are context dependent; safety and efficacy remain early-stage	Use dose-escalation, metabolic/pharmacodynamic monitoring, and biomarker-based patient selection
	LDHA	Converts pyruvate → lactate	FX11; GNE-140	Preclinical evidence, including breast cancer/TNBC models; no validated oncology clinical program	–	Potential disruption of normal lactate handling and limited tumor selectivity	Prioritize short-course or tumor-directed strategies and monitor lactate and acid-base status
	MCT1/MCT4	Lactate efflux and acidic-barrier maintenance; MCT4 may mediate bypass resistance	AZD3965	Phase I in advanced cancers; not TNBC-specific	NCT01791595	Lactate accumulation and acid-base disturbance may occur; MCT4 may mediate intrinsic or acquired resistance	Assess MCT1/MCT4 expression, monitor lactate, and consider sequential rather than prolonged blockade
FAO/OXPHOS (S2)	CPT1A	Gatekeeper for mitochondrial β-oxidation	Etomoxir (research use); emerging CPT1 inhibitors	TNBC/breast-cancer preclinical evidence; no validated TNBC clinical program for metabolic targeting	–	Hepatotoxicity, off-target mitochondrial effects, and interference with normal fatty-acid oxidation	Use research-grade inhibitors cautiously; monitor liver function and prioritize more selective next-generation agents
	NAMPT	Maintains NAD^+^ to support OXPHOS/Sirtuins	KPT-9274 (PAK4/NAMPT dual inhibitor)	Phase I in advanced solid tumors and non-Hodgkin lymphoma; no TNBC-specific clinical efficacy	NCT02702492	Systemic NAD^+^ depletion, potential myelosuppression and gastrointestinal toxicity; the dual-target mechanism complicates attribution	Consider intermittent dosing, complete blood-count and metabolic monitoring, and biomarker-enriched cohorts
	Complex I	Core component of OXPHOS	IACS-010759	Phase I in relapsed/refractory AML and in advanced solid tumors/lymphoma; development was terminated; not TNBC-specific	NCT02882321; NCT03291938	Very narrow therapeutic window, elevated lactate/lactic acidosis, vomiting, and peripheral neuropathy	Close lactate, blood-pH, and neurologic monitoring; short/intermittent schedules and stringent biomarker selection would be required
NADPH/one-carbon (S3)	G6PD (PPP)	NADPH generation and nucleotide biosynthesis	DHEA	Phase I/II in metastatic or unresectable synovial sarcoma; TNBC evidence remains preclinical	NCT02683148	Limited target selectivity, endocrine/steroid-related effects for DHEA, and potential disruption of normal redox homeostasis	Develop more selective agents, use time-limited exposure, and monitor hematologic and metabolic effects
	ME1	NADPH compensation	AS1134900	Preclinical evidence in cancer models; TNBC-specific clinical validation is lacking	–	Early-stage pharmacological development; systemic inhibition may affect normal NADPH and lipid metabolism; no established clinical safety profile	Develop tumor-selective inhibitors, use biomarker-based patient selection, and monitor redox and metabolic toxicity
	PHGDH/SHMT	Ser/Gly–one-carbon units for nucleotide synthesis	PHGDH inhibitors; SHMT inhibitors	TNBC-specific preclinical evidence; no validated oncology clinical program for metabolic targeting	–	Potential effects on normal proliferative and hematopoietic tissues; pharmacologic selectivity remains limited	Use biomarker selection for PHGDH/SGOC dependence, complete blood-count monitoring, and time-limited combinations
	xCT/SLC7A11	Cystine import to sustain GSH	Sulfasalazine	TNBC preclinical evidence; Phase I in recurrent glioblastoma and Phase III in metastatic colorectal cancer; no TNBC clinical efficacy	NCT04205357; NCT06134388	Gastrointestinal intolerance, hypersensitivity, and limited xCT selectivity; immune effects may be context dependent	Monitor gastrointestinal and hematologic toxicity and incorporate exposure and target-engagement biomarkers
Lipogenesis/cholesterol (S4)	ACLY/ACSS2	Acetyl-CoA supply/acetate utilization	Bempedoic acid (ACLY; non-oncology); MTB-9655 (ACSS2)	Phase III cardiovascular/non-oncology experience for bempedoic acid; Phase I in advanced solid tumors for MTB-9655; no TNBC efficacy	NCT02991118; NCT04990739	Non-oncology evidence cannot be extrapolated to TNBC; systemic lipid-metabolism perturbation and limited ACSS2 safety data remain concerns	Clearly separate oncology from non-oncology evidence; monitor liver/metabolic parameters and use biomarker-based enrollment
	FASN	*De novo* lipogenesis	TVB-2640 (FASN inhibitor)	Phase II in KRAS-mutant NSCLC and recurrent high-grade astrocytoma; TNBC evidence remains mainly preclinical	NCT03808558; NCT03032484	Dermatologic and ocular toxicity; no TNBC-specific clinical efficacy has been established	Monitor dermatologic and ocular adverse events; use biomarker-based selection for lipid-dependent tumors
	ACC1/ACC2	*De novo* lipogenesis	GS-0976 (ACC inhibitor)	Phase II clinical evidence in NASH; antitumor and TNBC evidence remains preclinical	NCT02856555; NCT03449446	The available clinical evidence is derived from a non-oncology indication; systemic lipid-metabolism disturbance and hypertriglyceridemia may limit translation to cancer therapy	Monitor plasma lipids and liver function; oncology-specific dosing, safety, and biomarker selection require further investigation
	SCD1	MUFA production; anti-ferroptosis	MTI-301	First-in-human Phase I study in metastatic, unresectable, or refractory solid cancers; TNBC is among relevant disease settings	NCT06911008	Safety, therapeutic window, and antitumor efficacy have not yet been established	Apply dose-escalation monitoring with lipidomic and pharmacodynamic assessment
Ferroptosis (S5)	GPX4	Detoxifies lipid peroxides	RSL3 (tool compound); preclinical candidates	TNBC and pan-cancer preclinical evidence only.	–	Potential severe normal-tissue toxicity from systemic GPX4 inhibition; RSL3 is not a clinical candidate	Prioritize tumor-selective or local delivery, short-window scheduling, and rigorous normal-tissue safety assessment
	FSP1/CoQ10	Plasma-membrane reductive system	FSP1 inhibitors in development	Preclinical evidence only	–	Selectivity, pharmacokinetics, and systemic lipid-redox effects remain unresolved	Develop selective compounds and pharmacodynamic biomarkers before pursuing combination strategies
	DHODH	Mitochondrial pyrimidine synthesis and lipid peroxidation control	Brequinar	TNBC-specific preclinical rationale; Phase Ib/IIa in relapsed/refractory AML; no TNBC clinical validation	NCT03760666	Myelosuppression, immunosuppression, and effects on normal proliferating cells	Monitor complete blood counts and infection risk, use short-course dosing, and carefully sequence with immunotherapy

## 3 Contextualizing S1–S5 Operating States within Established TNBC Classifications

The following discussion should be interpreted as a hypothesis-generating contextualization rather than a definitive one-to-one mapping. The S1–S5 framework describes dynamic metabolic operating states, whereas Lehmann [134,135] and Fudan classifications represent established molecular or transcriptomic stratification systems. Therefore, the potential enrichment of specific operating states within individual TNBC subtypes should be considered provisional and requires validation using standardized transcriptomic, metabolomic, spatial, and longitudinal cohorts.

### 3.1 Lehmann Classification

Lehmann and colleagues proposed six TNBC subtypes: two basal-like subtypes (BL1 and BL2), a mesenchymal subtype (M), a mesenchymal stem-like subtype (MSL), an immunomodulatory subtype (IM), and the LAR subtype. Subsequent work using quantitative histopathology and laser-capture microdissection indicated that the IM and MSL subtypes largely reflect tumors enriched for infiltrating lymphocytes and tumor-associated mesenchymal cells, respectively, refining the original scheme into a four-subtype TNBC classification (BL1, BL2, M, and LAR) [134,135].

### 3.2 Fudan Classification

Using transcriptomic and genomic analyses, the Fudan group further stratified TNBC into three distinct metabolomic subgroups: C1, characterized by enrichment of ceramides and fatty acids; C2, marked by elevated metabolites associated with oxidative reactions and glycosyltransferases; and C3, exhibiting the lowest degree of metabolic dysregulation. The LAR subtype overlaps with metabolomic C1, whereas the transcriptomic basal-like immune-suppressed (BLIS) subtype contains two prognostically distinct metabolomic subgroups, C2 and C6 [135].

### 3.3 Mapping Lehmann and Fudan Subtypes onto Metabolic States

#### 3.3.1 S1-Dominant State: BL1 and BLIS

The BL1 subtype is characterized by high Ki67 proliferation indices and aberrant activation of DNA damage response pathways [134]. Such extreme proliferative demand forces cells toward S1, driven by the HIF-1α-MYC axis and the Warburg program [10,13]. High concentrations of lactate produced by hyper-glycolysis can directly remodel chromatin, thereby stabilizing the gene-expression program of S1. Moreover, lactate in the tumor microenvironment can be imported by Treg cells via MCT1 and used as fuel to strengthen their immunosuppressive function [18,21], generating immune “cold” niches. This is consistent with features of the BLIS subtype [135].

#### 3.3.2 S2-Dominant State: M

The M subtype shows increased expression of EMT and growth-factor pathways, is often chemotherapy-insensitive, and exhibits high relapse rates [134], resembling DTPs commonly observed after chemotherapy [32,48]. Phase I trial data indicate that complex I inhibition (IACS-010759) in OXPHOS-high tumors can significantly suppress tumor growth, providing molecular support for a close relationship between the M subtype and S2 metabolic vulnerabilities [50].

#### 3.3.3 S3-Dominant State: BL2

The BL2 subtype is enriched for growth-factor signaling and myoepithelial markers, often accompanied by activation of EGFR, MET, and related pathways [134], creating sustained ROS pressure that pushes cells into the S3 program [53,55]. Consistent with this, subsets of BL2-like TNBC cells exhibit dependencies on serine synthesis enzymes such as PHGDH and SHMT [60,61,88], and sertraline can block SHMT1/2 to induce oxidative stress [80,81].

#### 3.3.4 S4/S5-Dominant States: LAR

The LAR subtype is characterized by luminal gene-expression programs driven by the androgen receptor (AR) [134,135]; metabolically, it is dominated by highly active lipogenesis (S4) [89,91,92]. FASN-driven fatty-acid synthesis not only supports tumor growth but appears to be a prerequisite for TNBC brain metastasis [102,109,111], which may explain the subtype’s distinctive metastatic propensity.

At the same time, excessive reliance on lipids can precipitate ferroptotic susceptibility (S5). Multi-omics analyses indicate that LAR cells accumulate large amounts of PUFA and show exceptional sensitivity to GPX4 inhibitors. These findings further demonstrate that combining GPX4 inhibitors with ICB can elicit potent antitumor immunity in LAR models, establishing S5 as a subtype-specific therapeutic window for LAR tumors [127].

In summary, the S1–S5 metabolic state spectrum proposed here is not intended to impose a static taxonomy on TNBC. Instead, using the language of “functional priorities and flux redistribution”, we seek to consolidate metabolically diverse observations across models, platforms, and treatment contexts into an actionable, comparable, and falsifiable working framework. Our central premise is that, under the combined pressures of hypoxia, nutrient fluctuation, immune stress, and therapeutic selection, TNBC metabolic plasticity does not diverge without limit; rather, it tends to shuttle among a finite set of operating modes with well-defined adaptive purposes: rapid expansion coupled to an acidic niche (S1), mitochondrial-dependent persistence and energy homeostasis (S2), NADPH-centered reductive defense coupled to one-carbon metabolism (S3), membrane and lipid-raft remodeling for migratory adaptation (S4), and the ferroptotic boundary that emerges when defenses fail (S5).

Importantly, S states do not correspond to sharply bounded biological entities. Within a single tumor, multiple states may coexist spatially, and the same cell population may traverse a temporal sequence of states, with phenotypes jointly shaped by microenvironmental constraints and therapeutic perturbations. Accordingly, the key question is not “which state a tumor belongs to”, but rather “which pressures act where, and in what sequence, to drive state transitions”, and “which transitions reveal exploitable therapeutic vulnerabilities”. In this sense, the S-state framework provides an operational narrative scaffold for metabolism-immune crosstalk, spatial heterogeneity, and resistance evolution: it allows lactate-acid barriers, lipid-enriched niches, immune exclusion, and post-treatment residual metabolic phenotypes to be interpreted and predicted on a shared dynamic map.

We also acknowledge important limitations in the current evidence, including model-to-model differences and incomplete causal chains. Many studies remain correlative, and closed-loop evidence linking “valve nodes” to flux rewiring, immune ecology, and therapeutic outcomes remains insufficient. Thus, the S-state framework is best viewed as an open hypothesis generator, valued for testable predictions: (i) therapeutic pressure will preferentially enrich certain states and form spatially defined niches; (ii) inhibition of one state often elicits predictable compensatory transitions; (iii) truly translatable strategies should target transition paths rather than single pathways. Building on these premises, the next section focuses on mechanism, asking which valve-level nodes allocate carbon, nitrogen, lipid, and reducing power, and thereby determine whether states can be locked, bypassed, or pushed across a tipping point.

## 4 Valve-Level Mechanisms for Carbon, Nitrogen, Lipid, and Redox Flux Allocation

Moving from state description to control logic requires identifying flux-valve nodes that redirect carbon, nitrogen, lipid, and reducing-power allocation [5]. The S1–S5 framework describes functional metabolic operating states, but transitions among these states are not driven simply by global upregulation of entire pathways. Instead, they are biased by pressure-responsive enzymes, transporters, and regulatory modules that determine whether substrates are routed toward lactate production, mitochondrial oxidation, anaplerosis, one-carbon metabolism, NADPH regeneration, lipid remodeling, or ferroptosis-related damage. In this sense, flux-valve nodes act as control points for state selection and compensatory bypass rather than as isolated therapeutic targets [5,136].

**Figure 2:**
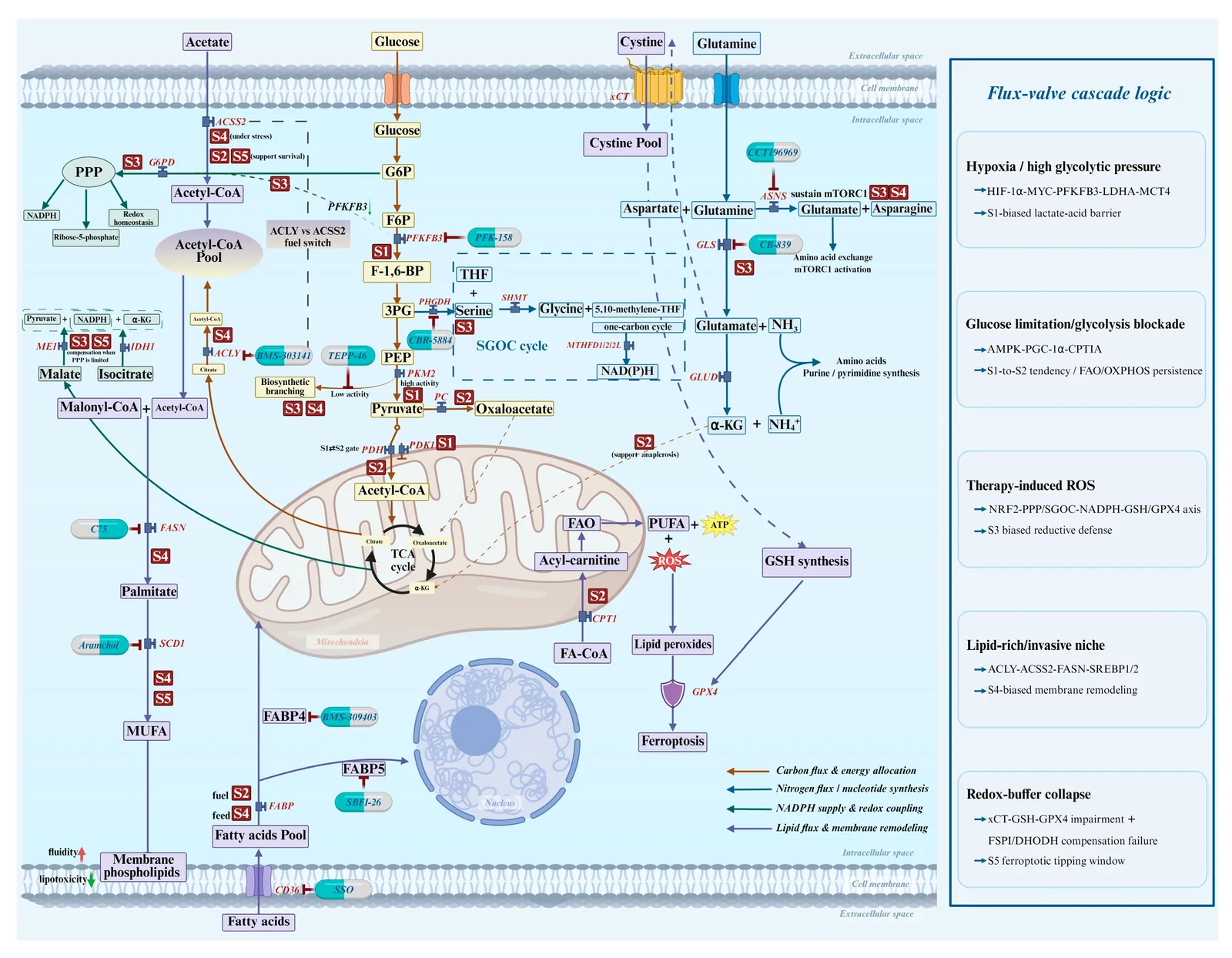
**Flux-valve map coupling carbon, nitrogen, lipid and redox allocation**. Integrated “flux-valve” schematic linking major nutrient inputs (glucose, acetate, glutamine, cystine, and fatty acids) to glycolysis/tricarboxylic acid (TCA), FAO/OXPHOS, one-carbon/nucleotide synthesis, lipid remodeling, and ferroptosis control. Key gatekeeper enzymes/transporters are positioned as control points for state selection and compensatory detours [e.g., ATP-citrate lyase (ACLY)/acyl-CoA synthetase short-chain family member 2 (ACSS2), Carnitine palmitoyltransferase 1 (CPT1), glucose-6-phosphate dehydrogenase (G6PD)/pentose phosphate pathway (PPP), PHGDH-SHM-MTHFD, and cystine/glutamate antiporter (xCT)/glutathione peroxidase 4 (GPX4)]. Colored arrows indicate coupled streams of carbon/energy routing, nitrogen flux for amino acid/nucleotide synthesis, NADPH/redox supply, and lipid flux for membrane remodeling. Created in BioRender.com.

Accordingly, this section summarizes four major flux-control axes: carbon and energy allocation, nitrogen and amino-acid allocation, lipid allocation, and NADPH/redox allocation. These axes are coupled through shared intermediates such as pyruvate, acetyl-CoA, glutamine-derived carbon and nitrogen, cystine, glutathione, and lipid peroxides [137]. This coupling explains why single-pathway inhibition often produces adaptive switching rather than durable tumor control [4,26,136]. For example, glycolytic blockade may promote FAO/OXPHOS-supported persistence; oxidative therapy may select for NADPH-dependent reductive defense; and lipid remodeling may either support membrane adaptation or prime ferroptotic vulnerability [113]. The translational value of the flux-valve concept therefore lies in predicting both the dominant metabolic state and the most likely escape route after therapeutic pressure [138] (Fig. 2).

### 4.1 Carbon-Flux Valves: From Glycolytic Expansion to Mitochondrial Persistence

Carbon-flux valves determine whether glucose-derived carbon is retained in rapid glycolysis, oxidized in mitochondria, diverted into biosynthesis, or converted into acetyl-CoA for lipid remodeling. In the S1 state, the HIF-1α-MYC-PFKFB3-LDHA-MCT4 axis favors high-throughput glycolysis, lactate production, and lactate export [9,17,19,20,139]. PFKFB3 increases glycolytic drive, LDHA converts pyruvate to lactate, and MCT4 exports lactate to maintain intracellular pH while constructing an extracellular acidic barrier. This carbon-routing pattern supports rapid proliferation and immune exclusion, but it also creates dependence on glucose supply and lactate-export capacity.

A central carbon decision point is the pyruvate-to-acetyl-CoA gate [137]. When pyruvate dehydrogenase activity is restrained by PDK-related signaling, pyruvate entry into the tricarboxylic acid cycle is limited and carbon is preferentially retained in the lactate-producing S1 program [26]. Conversely, when glucose availability becomes limited or S1 is therapeutically blocked, energy stress can activate AMPK and promote PGC-1α- and CPT1A-associated mitochondrial adaptation, shifting cells toward the S2 FAO/OXPHOS persistence state [30,33,34,35]. Therefore, S1 blockade may fail as monotherapy if it is not paired with strategies that anticipate mitochondrial bypass.

Carbon flux also feeds lipid remodeling through acetyl-CoA-generating valves. ACLY converts citrate into cytosolic acetyl-CoA, whereas ACSS2 allows acetate to substitute for glucose-derived acetyl-CoA under hypoxia or nutrient stress [89,93,97,100]. These valves connect carbon allocation to the S4 lipid-remodeling state, enabling fatty acid and cholesterol synthesis even when canonical glucose-derived carbon becomes constrained. Thus, carbon-flux valves link S1 expansion, S2 persistence, and S4 membrane remodeling through a shared resource-allocation logic.

### 4.2 Nitrogen and Amino-Acid Flux Valves: Anaplerosis, Nucleotide Supply, and Immune Pressure

Nitrogen-flux valves determine whether amino-acid-derived substrates support mitochondrial anaplerosis, nucleotide synthesis, redox defense, or immune suppression. Glutamine-related nodes, including GLS and GLUD, connect nitrogen metabolism to both carbon replenishment and antioxidant defense. Through glutamate and α-ketoglutarate production, glutamine metabolism can sustain the tricarboxylic acid cycle, support mitochondrial adaptation, and contribute to glutathione synthesis [70,140,141]. This makes glutamine flux particularly relevant to S2 persistence, S3 reductive defense, and S5 ferroptosis buffering.

Serine–glycine–one-carbon metabolism forms another nitrogen-linked control axis. The PHGDH–SHMT–MTHFD module diverts glycolytic intermediates into serine and one-carbon units, thereby supporting nucleotide synthesis, methylation reactions, and NADPH production [60,61,62,76,88]. Under therapy-induced oxidative stress, this module reinforces the S3 state by coupling biosynthetic repair to reductive defense. In this context, one-carbon metabolism is not merely a proliferation pathway; it becomes a survival valve that helps cells withstand chemotherapy, radiotherapy, and inflammatory stress.

Amino-acid flux also shapes immune ecology [142]. Competition for arginine, glutamine, and tryptophan can restrict effector T-cell function, while kynurenine production and myeloid-associated amino-acid depletion can reinforce immunosuppressive niches [142,143,144,145]. Therefore, nitrogen-flux valves help connect tumor-cell intrinsic survival programs with the metabolic fitness of infiltrating immune cells. This explains why amino-acid metabolism can simultaneously support tumor persistence and immune escape.

### 4.3 Lipid-Flux Valves: Membrane Remodeling, FAO, and Ferroptosis Priming

Lipid-flux valves determine whether fatty acids are oxidized for survival, synthesized for membrane remodeling, imported from the microenvironment, or incorporated into ferroptosis-sensitive phospholipids. In the S2 state, CPT1A controls mitochondrial entry of long-chain fatty acids and supports FAO/OXPHOS-dependent persistence [34,39,40]. This pathway is particularly important in nutrient-poor or post-treatment residual niches, where glycolytic expansion becomes less stable and mitochondrial energy maintenance becomes advantageous.

In contrast, the S4 state is driven by lipid synthesis and membrane-architecture remodeling. ACLY, ACSS2, ACC, FASN, SCD1, ACSL4, and SREBP1/2 collectively support fatty acid synthesis, cholesterol metabolism, lipid-droplet formation, and lipid-raft organization [89,96,102]. These changes influence receptor signaling, adhesion, migration, immune-checkpoint responsiveness, and drug uptake. Importantly, lipid remodeling can also create resistance to therapeutic agents by altering membrane composition or enabling compensatory lipid uptake through pathways such as CD36-mediated import.

The lipid axis also creates a bridge to S5. ACSL4-mediated incorporation of polyunsaturated fatty acids into phospholipids can increase membrane plasticity and metastatic behavior, but it also raises sensitivity to lipid peroxidation [102,106,117,118]. Conversely, SCD1-mediated monounsaturated fatty acid production may buffer ferroptosis by reducing the relative abundance of oxidation-prone membrane lipids [103,124]. Thus, lipid-flux valves place S4 and S5 in a dynamic relationship: the same membrane remodeling that supports invasion and resistance may also prime tumor cells for ferroptotic collapse when antioxidant defenses fail.

### 4.4 NADPH and Redox Valves: Reductive Defense Versus Ferroptotic Collapse

NADPH is a limited reducing currency shared by biosynthesis, antioxidant defense, and ferroptosis suppression. The S3 state is therefore governed by valves that regenerate and allocate NADPH. The pentose phosphate pathway, particularly G6PD-dependent flux, provides a major source of NADPH under oxidative stress. ME1/ME2 and IDH1/IDH2 provide complementary NADPH-generating routes, while the SGOC pathway couples one-carbon metabolism to nucleotide synthesis and reductive defense [53,56,119]. Together, these branches allow TNBC cells to maintain glutathione and thioredoxin systems during therapy-induced ROS stress.

The xCT/GSH/GPX4 axis represents the most direct redox valve linking S3 to S5. xCT imports cystine for glutathione synthesis, while GPX4 uses glutathione to detoxify lipid hydroperoxides [72,120,123]. When NADPH supply, cystine import, glutathione regeneration, or GPX4 activity becomes insufficient, lipid peroxides accumulate and the system shifts toward the S5 ferroptotic tipping point [117,119,123]. FSP1/CoQ10 and DHODH provide additional GPX4-bypass defense routes, but these compensatory systems may also become therapeutically targetable when S3-like redox buffering is exhausted [128,130].

This redox logic explains why ferroptosis induction is most rational as a timed strategy rather than nonspecific long-term redox disruption. Radiotherapy, platinum chemotherapy, and other ROS-inducing treatments may create transient oxidative windows during which S3 defenses are overloaded and S5 vulnerability becomes more accessible [117,120,146]. Therefore, the therapeutic goal is not simply to inhibit one antioxidant enzyme, but to identify when the tumor has become dependent on a narrow set of redox valves [128].

### 4.5 Cascade Logic of Flux-Valve-Driven S1–S5 Switching

The cascade relationship between flux-valve nodes and state switching can be summarized as follows. Hypoxia and high glycolytic pressure activate HIF-1α-MYC-PFKFB3-LDHA-MCT4 signaling, reinforcing the S1 glycolysis–lactate/acidity barrier [9,139]. Glucose limitation, intracellular acid stress, or glycolytic blockade can activate AMPK-PGC-1α-CPT1A signaling, promoting an S1-to-S2 shift toward FAO/OXPHOS-supported persistence [26,35]. Therapy-induced ROS engages NRF2-, PPP-, SGOC-, NADPH-, GSH-, and GPX4-related valves, reinforcing the S3 reductive-defense state [53,75]. Lipid-rich, invasive, or metastatic niches activate ACLY/ACSS2–FASN–SCD1–SREBP1/2-centered lipid flux, supporting the S4 membrane-remodeling state [89,102]. Finally, when xCT/GSH/GPX4-centered defense fails and FSP1/DHODH compensation becomes insufficient, lipid peroxides accumulate and cells may cross the S5 ferroptotic tipping point [117,125,128].

This cascade model helps explain why TNBC metabolic adaptation is predictable despite its apparent heterogeneity. Each therapeutic or microenvironmental pressure does not randomly reshape metabolism; rather, it biases flux through a limited set of valve nodes and thereby enriches particular operating states or escape routes. Clinically, this suggests that metabolism-targeted strategies should not only suppress a dominant state, but also anticipate the next compensatory transition. In this sense, flux valves provide a mechanistic bridge between metabolic state mapping, resistance prediction, and temporally staged combination therapy, as summarized in Table 2.

**Table 2:** Flux-switch matrix.

Flux Axis	Core Switch	Function	State Impact	Investigational/Approved Agents	Phase	Trial ID
Carbon flux/energy allocation	PFKFB3	Glycolysis accelerator	Locks in S1	PFK-158	I	NCT02044861
	PKM2	Dimer → shunting; Tetramer → lactate	Intra-S1 tuning	TP-1454	I	NCT04328740
	PDK1–PDH	Gatekeeping mitochondrial entry	S1 ↔ S2/TCA switching	Dichloroacetate	II	NCT01386632
	PC vs. GLS	Anaplerotic source (pyruvate vs. glutamine)	Fuel choice for S2/TCA	IACS-6274 CB-839	III	NCT05039801; NCT03057600
	ACLY vs. ACSS2	Acetyl-CoA source (citrate vs. acetate)	Precursor switching toward S4	MTB-9655	I	NCT04990739
Nitrogen flux/nucleotides	GLS/GLUD	Glutamine → glutamate flux	GSH supply in S3/S5	Preclinical	–	–
	PHGDH/SHMT	Ser/Gly → one-carbon units	One-carbon defense in S3	–	–	–
	MTHFD1/2	One-carbon oxidation → NADPH/nucleotides	Core of S3	TH9619	I/II	–
NADPH supply	G6PD	PPP rate-limiting step	High-flux valve for S3	DHEA	I	NCT02683148
	ME1/ME2	Malate → pyruvate + NADPH	Complementary route for S3	–	–	–
	IDH1/IDH2	Isocitrate → α-KG + NADPH	Coupling between S3 and TCA	AG-120; AG-221	II	NCT02074839; NCT02273739
Lipid flux	CD36/FABP	Fatty-acid uptake	Substrate supply for FAO in S2	–	–	–
	CPT1	Rate-limiting entry to FAO	Core switch for S2	–	–	–
	ACC	Inhibits CPT1; promotes lipogenesis	Trade-off between S2 ↔ S4	GS-0976; PF-05221304	II	NCT02856555; NCT03248882
	FASN	*De novo* lipogenesis	Core of S4	TVB-2640	I	NCT02223247
	SCD1	Saturated FA → MUFA	Membrane-lipid tuning in S4/S5	MTI-301	I	NCT06911008
Redox/ferroptosis	xCT	Uptake of GSH precursors	First-line defense in S5	Sulfasalazine	I/II	NCT05580861
	GPX4	Detoxification of lipid peroxides	Gatekeeper of S5	–	–	–
	FSP1/DHODH	GPX4-bypass resistance	Redundant protection in S5	Brequinar PTC299 BAY2402234	Ib/IIa	NCT03760666; NCT03761069; NCT03404726

## 5 Metabolic Reprogramming in the Tumor Microenvironment: Metabolites as Hubs

**Figure 3:**
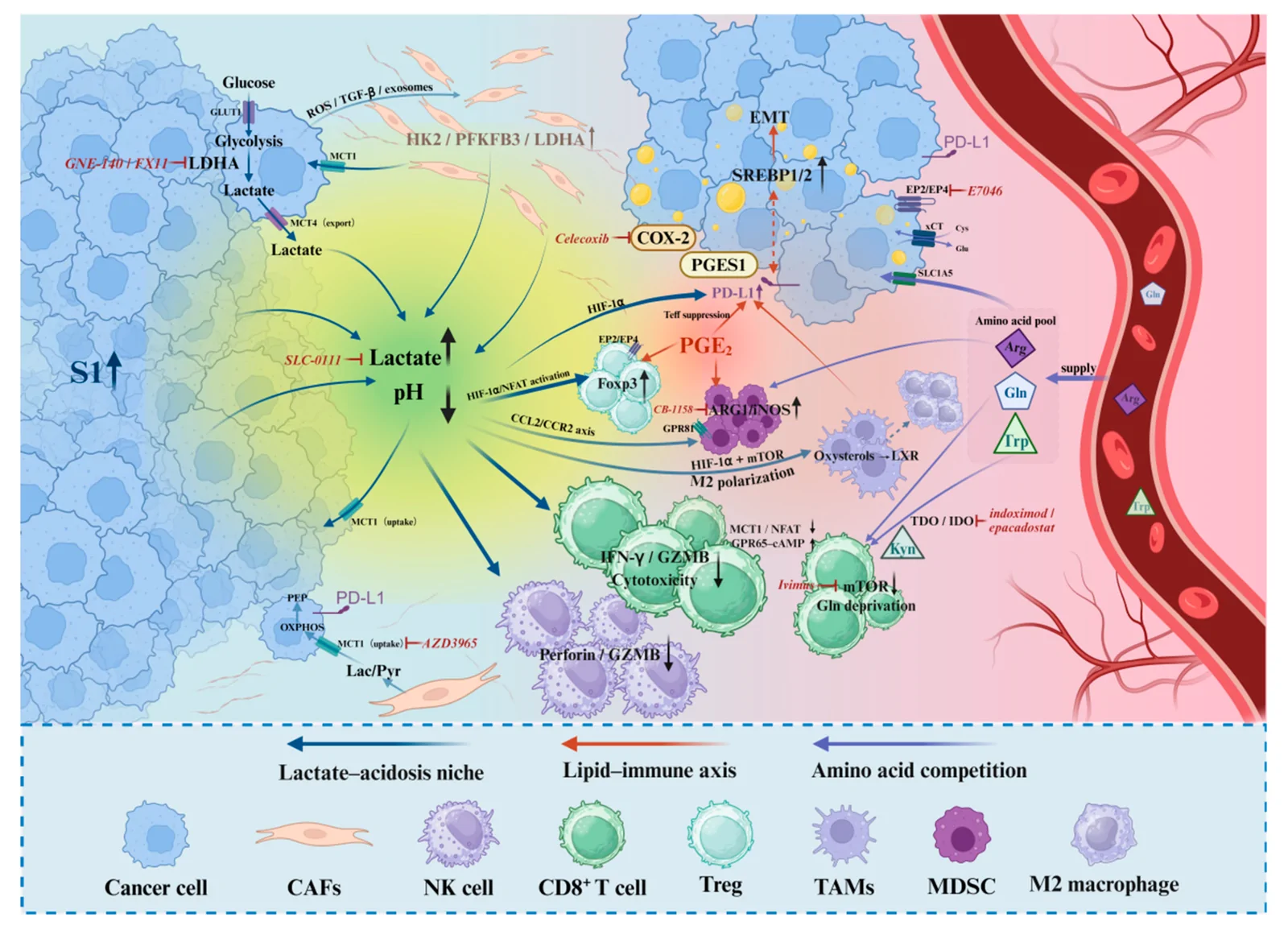
**Metabolic barrier niches and immune-cell-intrinsic metabolic adaptation in the TNBC tumor microenvironment**. Spatial metabolic isozones shape immune exclusion through lactate–acidosis, lipid–immune signaling, and amino-acid competition. S1-associated lactate export and acidosis impair CD8^+^ T-cell glycolysis, cytokine production, and cytotoxic activity, whereas S4-associated lipid remodeling, cholesterol/lipid-raft signaling, cyclooxygenase 2 (COX-2)/prostaglandin E2 (PGE2), and Programmed death-ligand 1 (PD-L1)-related pathways promote immune tolerance and immune checkpoint blockade (ICB) resistance. In addition, different isozones may reshape immune-cell-intrinsic metabolism: CD8^+^ effector T cells require glucose, mitochondrial fitness, and amino-acid supply, whereas Tregs, tumor-associated macrophages (TAMs) and myeloid-derived suppressor cells (MDSCs) may be favored in lactate-rich, lipid-enriched, redox-buffered, or FAO-biased niches. Created in BioRender.com.

In the preceding sections, we focused on the cell-intrinsic metabolic rewiring networks of TNBC cells. However, these metabolic programs do not operate in isolation. TNBC cells continuously release or consume metabolites, including lactate, lipids, glutamine, arginine, and tryptophan-related metabolites, thereby reshaping the TME and constructing layered metabolic barriers that support immune exclusion, effector dysfunction, and therapeutic resistance [18,21,89,91,94]. In this section, we summarize how S-state-associated metabolites act as hubs of tumor–immune crosstalk rather than passive metabolic by-products (Fig. 3) [112,113,114,144].

### 5.1 Lactate-Acidosis Niche: External Consequences of S1

The S1 state creates a prototypical lactate-acidic barrier. In S1-dominated TNBC territories, high glycolytic flux and lactate export increase extracellular lactate and lower pH, forming a “chemical trench” around hypoxic or highly glycolytic tumor regions [18,21]. This niche suppresses effector immunity through several linked mechanisms. Lactate and acidosis impair T-cell glycolysis, reduce cytotoxic cytokine production, and weaken CD8^+^ T-cell and NK-cell effector function [18,21]. Lactate can also promote myeloid-derived suppressor cell (MDSC) recruitment and TAM polarization, thereby amplifying local immunosuppression [27,28,147]. In addition, lactate-related signaling can cooperate with PD-L1 and other immune-checkpoint programs, converting metabolic suppression into a more stable immune-evasion phenotype [29,148].

From the perspective of the S-state framework, lactate is therefore not merely a glycolytic waste product [149]. It is a niche-engineering molecule that links S1 dominance to immune exclusion, myeloid suppression, and reduced sensitivity to immune checkpoint blockade [18,21,27,28]. This explains why S1-targeted therapy should not only suppress glycolysis or lactate export, but also aim to reopen immune access to the tumor bed.

### 5.2 Lipid-Cholesterol Niche: External Consequences of S4

The S4 state establishes a lipid-enriched and cholesterol-remodeled immune barrier, particularly at invasive fronts and metastatic niches. Tumor cells and inflammatory CAFs can accumulate lipid droplets and cholesteryl esters, creating a lipid-rich region that favors invasion and immunosuppression [91,94]. Lipid mediators such as prostaglandin E2 (PGE2) can suppress T-cell and NK-cell activation [150] and promote PD-L1 expression, whereas cholesterol-derived oxysterols can activate liver X receptor (LXR) signaling, reduce CD8^+^ T-cell abundance or promote exhaustion, and favor Treg or M2-like macrophage programs [151].

Thus, the S4 niche does not only provide structural lipids for tumor-cell membranes. It also remodels immune-cell signaling thresholds and stabilizes an immunosuppressive lipid–immune axis [91,94,112,113,114]. Therapeutically, this supports the rationale for combining lipid-remodeling interventions with ICB or drug-delivery strategies, especially in tumors with lipid-rich invasive fronts or membrane-remodeling features [115,116].

### 5.3 Amino-Acid Competition and Nutrient-Depletion Niches

Amino-acid metabolism provides another route by which TNBC suppresses antitumor immunity. Glutamine and arginine consumption can create local nutrient-depletion zones that overlap with S2/S3-like metabolic territories. TNBC cells with high glutamine uptake can deprive effector T cells of substrates needed for mTORC1 activation, proliferation, and differentiation, thereby promoting dysfunctional or exhausted T-cell phenotypes [142,144]. Similarly, ARG1-mediated arginine depletion can impair T-cell receptor signaling and effector maintenance, so that immune cells entering the tumor fail to sustain cytotoxic activity [78,79].

Tryptophan metabolism may further reinforce this suppressive amino-acid landscape through kynurenine-related immune regulation [144]. Together, glutamine, arginine, and tryptophan competition indicate that immune exclusion is not only a problem of physical infiltration, but also a problem of metabolic fitness. Beyond metabolite-mediated immune suppression, these metabolic isozones may reshape immune-cell-intrinsic metabolism [152]: CD8^+^ effector T cells depend on glucose availability, mitochondrial fitness, and amino-acid supply, whereas Tregs and suppressive myeloid cells may better tolerate lactate-rich, lipid-enriched, redox-buffered, or FAO-biased niches [77,152].

### 5.4 CAFs, Matrix Densification, and Stabilization of Metabolic Barriers

Non-immune stromal components, especially CAFs and the ECM, further stabilize metabolic heterogeneity. CAFs promote collagen deposition, matrix crosslinking, tissue stiffness, and vascular compression, thereby restricting oxygen and nutrient diffusion and maintaining persistent hypoxic, acidic, and nutrient-depleted territories [153,154,155]. These physical constraints reinforce local S-state selection and help maintain spatial metabolic barriers [18,21].

Different CAF subsets may contribute to this process through distinct mechanisms. Myofibroblastic CAFs (myCAFs) are linked to TGF-β-driven matrix deposition and mechanical remodeling, whereas inflammatory CAFs (iCAFs) can secrete cytokines and accumulate lipid droplets, supporting inflammatory and lipid-rich niches [91,94,112,113,114]. Antigen-presenting CAFs (apCAFs) may express MHC class II molecules but lack sufficient co-stimulatory capacity, potentially contributing to ineffective T-cell activation. Overall, CAFs create a dual barrier: a physical barrier that limits immune trafficking and a metabolic barrier that reshapes oxygen, nutrient, lactate, and lipid gradients.

In summary, metabolic reprogramming in TNBC is not merely an energy engine for tumor cell proliferation, but a proactive strategy to remodel the immune microenvironment and erect defensive barriers. From the “acidic shield” built by an S1 program, to the “lipid trap” laid by S4, and the “nutrient desert” engineered by S2/S3, metabolic phenotypes and immunosuppressive mechanisms show a striking internal coherence [18,21,91,94]. This implies that metabolic heterogeneity directly shapes the spatial immune landscape of tumors. Consequently, treating metabolic features as a new class of “immune checkpoints” has become an emerging focus; implementing precision, subtype-aware strategies to dismantle metabolic barriers may represent a key route to overcoming resistance to immunotherapy in TNBC [112,113,114,115].

## 6 Therapy-Induced Metabolic Plasticity and DTPs

**Figure 4:**
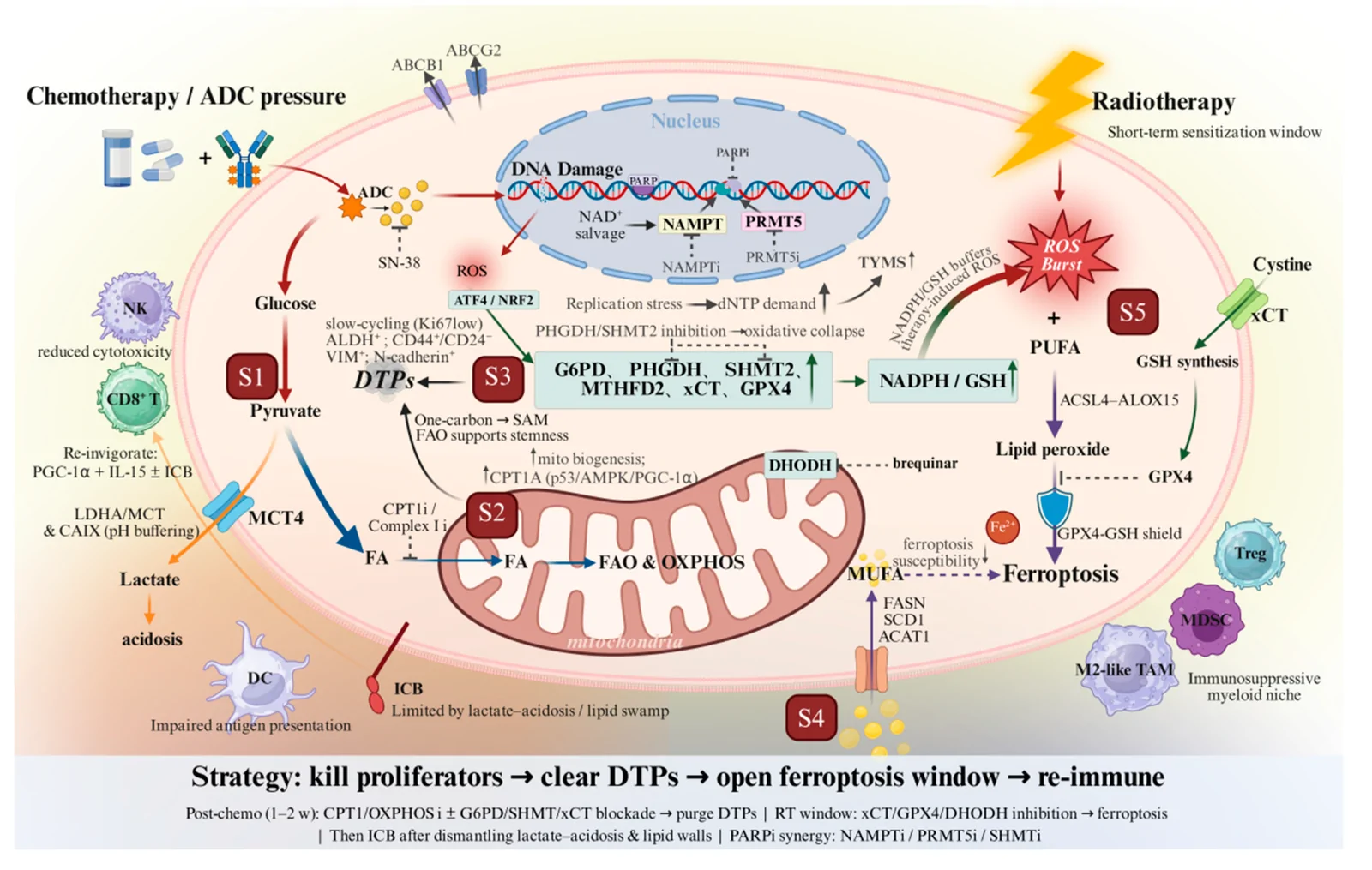
**Therapy-induced remodeling and timed vulnerability windows**. Schematic illustrating how chemotherapy/antibody-drug conjugate (ADC) pressure and radiotherapy reshape metabolic states and generate drug-tolerant persisters (DTPs). Cytotoxic pressure preferentially removes proliferative programs, while residual cells enrich stress-defense and persistence modules (e.g., FAO/OXPHOS and NADPH/GSH buffering) and engage DNA damage–associated salvage pathways. Radiotherapy-associated ROS bursts are depicted as a short sensitization window that can be exploited by weakening antioxidant/ferroptosis defense (xCT/GPX4 and related nodes) to promote lipid peroxidation and ferroptosis. The bottom line summarizes a sequence-aware strategy: debulk proliferators → purge DTPs → open a ferroptosis window → restore antitumor immunity. Created in BioRender.com.

Therapy not only eliminates tumor cells but also powerfully perturbs the metabolic landscape, driving state reconfiguration and selectively enriching persistence-prone subclones [156]. In this section, we discuss how chemotherapy [39,40], radiotherapy [53], and PARP inhibitors (PARPi) induce metabolic adaptation in TNBC, and how therapy-induced metabolic liabilities can be therapeutically exploited (Fig. 4) [48,157].

### 6.1 Chemotherapy- and ADC-Induced S1-to-S2/S3 Remodeling

Conventional chemotherapy and ADC-based cytotoxic pressure preferentially eliminate rapidly proliferating, glycolysis-biased S1-like tumor cells [158]. However, residual TNBC cells frequently display a shift toward mitochondrial adaptation and redox defense. One recurrent route is S1-to-S2 switching, in which surviving cells increase FAO/OXPHOS dependence, mitochondrial fitness, and anti-apoptotic buffering. This phenotype supports slow-cycling persistence rather than rapid expansion. Another route is S1-to-S3 or S2-to-S3 reinforcement, in which therapy-induced oxidative stress selects for NADPH/GSH/GPX4-dependent antioxidant defense [159]. Together, these changes allow DTPs to tolerate DNA damage, oxidative pressure, and nutrient stress [47,53,158].

This logic explains why single metabolic inhibitors often show limited durability [136]. Blocking glycolysis may select for mitochondrial bypass; inhibiting mitochondrial respiration may increase dependence on redox buffering; and disrupting redox defense may become effective only when oxidative pressure is already high. Therefore, chemotherapy- or ADC-induced residual disease may be more vulnerable to staged combinations that first debulk proliferative S1-like populations and then target S2/S3-like persistence programs [26,50,75,77].

### 6.2 Radiotherapy-Induced Oxidative Windows and S3-to-S5 Vulnerability

Radiotherapy creates a transient oxidative burst that can push TNBC cells toward either survival or collapse [146]. If antioxidant capacity remains sufficient, tumor cells reinforce the S3 reductive-defense state through NRF2-related signaling, NADPH regeneration, glutathione recycling, and GPX4-dependent lipid-peroxide detoxification. This adaptation can protect residual cells from radiation-induced damage and contribute to treatment resistance [53,56,123].

However, the same oxidative pressure may also create a therapeutic window. When NADPH supply, cystine import, GSH regeneration, or GPX4 activity becomes insufficient, lipid peroxides accumulate and cells may cross the S5 ferroptotic tipping point. Thus, radiotherapy-associated oxidative stress may be exploited by timed inhibition of xCT/GSH/GPX4-related defenses, DHODH-dependent compensation, or other ferroptosis-suppressive systems. The key is timing: ferroptosis-oriented strategies are most rational during a short oxidative window rather than as nonspecific long-term redox disruption [117,121,146].

### 6.3 ICB-Associated Metabolic Selection and Immune-Access Remodeling

ICB can reshape tumor metabolism by altering immune pressure within the TME. Responding tumors may show reduced lactate–acidity barriers, improved CD8^+^ T-cell infiltration, and partial restoration of immune effector function. In contrast, non-responding tumors may maintain S1-like acidic exclusion, S4-like lipid-rich immune tolerance, or S3-like redox buffering that protects both tumor cells and suppressive immune programs. Therefore, resistance to ICB may be maintained not only by canonical immune-checkpoint pathways, but also by persistent metabolic barriers that prevent immune access or impair immune-cell metabolic fitness [18,21,27,91,94].

In this context, metabolism-targeted therapy should not be viewed as a replacement for immunotherapy, but as a way to remodel immune accessibility. Strategies that reduce lactate export, alleviate acidosis [21,27], weaken lipid-mediated immune tolerance [91,112], or disrupt amino-acid competition [142,144] may help convert metabolically protected niches into more permissive immune territories. However, such combinations require biomarker-guided selection because the dominant barrier may differ across tumor regions and treatment stages [18,27,91,112].

### 6.4 PARPi-Associated Metabolic Compensation

PARPi induces DNA damage and replication stress, but residual TNBC cells may survive by activating compensatory metabolic programs. These include NADPH-dependent redox buffering, one-carbon metabolism for nucleotide repair and methylation support [56,119], and mitochondrial adaptation that sustains energy balance under genotoxic stress [47]. Thus, PARPi resistance may involve both DNA-repair pathway adaptation and metabolic compensation.

From the S-state perspective, PARPi-associated resistance is particularly linked to S3-like reductive defense and, in some contexts, S2-like mitochondrial persistence. This suggests that PARPi combinations may benefit from selectively weakening NADPH/one-carbon support [56,119], NAMPT-related stress adaptation [44,84], or mitochondrial survival programs [47]. Such strategies should be interpreted as hypothesis-generating rather than clinically established, because many of these pathways also support normal proliferating and immune cells.

### 6.5 Shared Metabolic Resistance Routes

Across chemotherapy, ADCs, radiotherapy, ICB, and PARPi, DTPs repeatedly use several shared metabolic resistance routes. First, flux bypass allows tumor cells to escape single-pathway blockade, such as shifting from glycolysis inhibition toward FAO/OXPHOS-supported survival [39,47,160]. Second, redox compensation enables cells exposed to chemotherapy or radiotherapy to reinforce NRF2-, NADPH-, GSH-, and GPX4-dependent antioxidant defenses [53,55,57]. Third, nutrient scavenging programs, including autophagy, macropinocytosis, acetate utilization, glutamine rewiring, and exogenous lipid uptake, provide alternative substrates when canonical nutrient routes are restricted [93,161]. Fourth, lipid and membrane remodeling alters receptor signaling, drug uptake, lipid-raft organization [102,103], and immune-checkpoint responsiveness [112,162]. Finally, persister maintenance programs combine low proliferation, mitochondrial dependence, anti-apoptotic buffering, and stress-adaptive signaling to preserve residual tumor cells after treatment [39,40,47,160].

These shared routes explain why targeting one metabolic pathway is rarely sufficient [163]. A more rational strategy is to identify the dominant therapy-induced state and simultaneously anticipate the most likely escape route. In practical terms, this supports a sequence-aware intervention logic: first debulk proliferative tumor cells, then target S2/S3-like persister survival, exploit transient S5 ferroptosis windows when oxidative stress is high, and finally maintain pressure against compensatory bypass [164]. This approach keeps the S1–S5 framework aligned with therapeutic timing rather than treating metabolic states as fixed tumor categories.

## 7 Spatial Ecology and Two-Tier Monitoring of S Metabolic States

**Figure 5:**
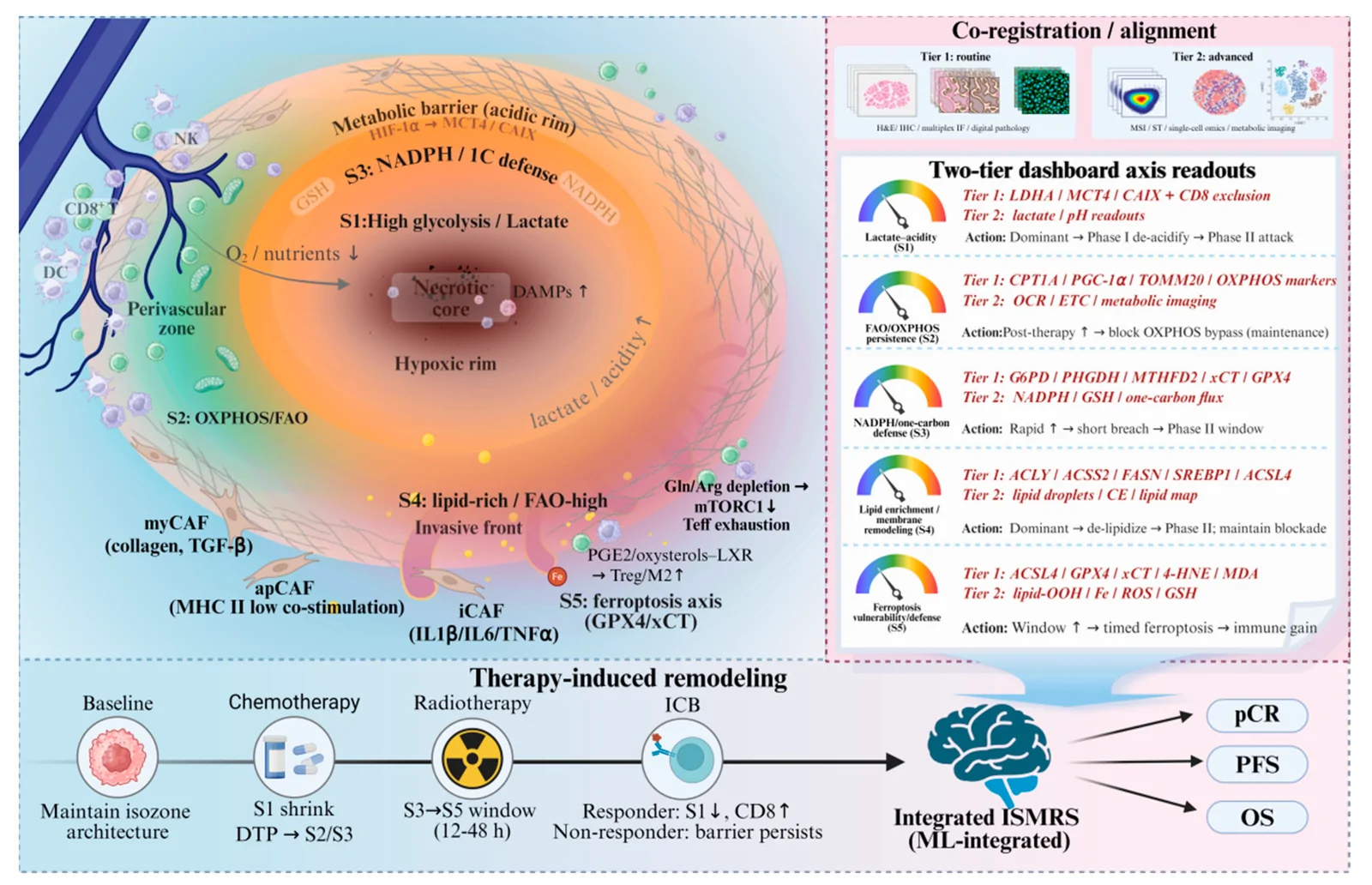
**Spatial metabolic isozones and two-tier dashboard axis readouts for TNBC state monitoring**. The left panel depicts spatial isozones associated with lactate-acidity barriers, FAO/OXPHOS-enriched persistence, NADPH/one-carbon defense, lipid-rich invasive fronts, and ferroptosis vulnerability. The right panel summarizes Tier 1 routine pathology readouts, including hematoxylin and eosin (H&E), immunohistochemistry (IHC), multiplex immunofluorescence (IF), and digital pathology, and Tier 2 advanced profiling, including mass spectrometry imaging (MSI), spatial transcriptomics (ST), single-cell omics, metabolic imaging, oxygen consumption rate (OCR), electron transport chain (ETC) assessment, lactate/pH mapping, lipid profiling, and redox-related readouts. Created in BioRender.com.

The S1–S5 framework becomes more clinically useful when metabolic states are interpreted spatially rather than as bulk tumor averages [165]. Classical studies of cancer metabolism have largely relied on bulk biochemical assays of tumor specimens or *in vitro* cell lines, implicitly assuming metabolic homogeneity within tumors or compressing heterogeneity into discrete subclones. TNBC, however, is a highly organized three-dimensional ecosystem, whose metabolic landscape is co-sculpted by vascular architecture, oxygen gradients, nutrient availability, matrix densification and the spatial distribution of cellular lineages. Recent spatial omics and metabolic imaging approaches now make it possible to resolve metabolites, metabolic enzymes, transcriptional programs, and immune-cell neighborhoods *in situ* [166]. Within this context, metabolic “isozones” can be understood as spatial territories that share similar substrate availability, stress intensity, stromal architecture, and immune accessibility (Fig. 5) [4,5,6,7].

### 7.1 Prototypical Metabolic Isozones

Based on vascular proximity, oxygen tension, nutrient supply, matrix pressure, and immune infiltration, TNBC can be simplified into three prototypical metabolic isozones. These zones are not fixed anatomical compartments, but recurring spatial patterns that help explain how S states coexist and switch within the same tumor [4,7,165].

Zone A, the hypoxic or peri-necrotic core, is commonly enriched for S1-like lactate–acidity features [18,20,167]. Severe hypoxia stabilizes HIF-1α, promotes glycolytic flux, and increases lactate export through MCT4 and acid regulation through CAIX. The resulting high-lactate and low-pH niche can limit CD8^+^ T-cell function, promote Treg or myeloid suppressive activity, and establish an immune-cold territory [21,168]. Therefore, Zone A is best interpreted as a lactate–acid barrier rather than simply a poorly perfused tumor core [18,20,21].

Zone B, the vessel-rich rim, receives more oxygen and circulating nutrients, including glucose, glutamine, and fatty acids. In this region, tumor cells may display mixed S2/S4 tendencies [45,46,89], with mitochondrial activity, mTORC1/c-MYC-related growth signals, and lipid remodeling coexisting to different degrees. Immune cells may enter this region more easily, but local nutrient competition, PD-L1 induction, or IDO1-related tryptophan metabolism can still drive functional exhaustion [94,145].

Zone C, the invasive front, frequently shows lipid-enriched and matrix-remodeled features consistent with S4-like behavior [89,102]. Tumor cells interact with CAFs, adipocytes, and extracellular matrix components, while CD-mediated fatty-acid uptake, FASN activity, and ACSL4-related membrane remodeling support invasion, EMT, and metastatic dissemination [102,106,113]. Thus, Zone C often functions as both a metastatic launchpad and an immune/metabolic barrier.

### 7.2 Multimodal Spatial Profiling

Spatial metabolic states cannot be reliably assigned by a single marker [165,169]. Instead, S-state tendencies should be inferred from convergent evidence across morphology, protein markers, metabolite distributions, transcriptional programs, and immune-cell context. MSI provides metabolite-layer evidence by mapping lactate, fatty acids, acylcarnitines, lipid species, and other metabolites directly on tissue sections [153]. ST provides program-level evidence by mapping glycolysis, OXPHOS, FAO, antioxidant-response, one-carbon, and lipid-remodeling gene signatures to spatial coordinates [165]. IHC and multiplex IF provide routine-accessible anchors, including markers such as MCT4, CAIX, LDHA [17,19,20], CPT1A [37], G6PD, PHGDH, GPX4 [63,69,71], FASN, SCD1, ACSL4 [102,103,106], CD8, PD-L1, and CAF markers [169].

A practical workflow is therefore based on co-registration. H&E provides the morphological basemap; IHC/IF defines protein and immune-cell landmarks; MSI adds chemical localization; and ST links metabolite gradients to transcriptional programs and cellular composition. This multimodal registration strategy does not convert S1–S5 into fixed diagnostic subtypes [170]. Rather, it supports spatial inference of dominant barriers, mixed states, and transition corridors, such as rim-to-core gradients, invasive-front lipid enrichment, or immune-infiltrated versus immune-excluded regions [4,7].

### 7.3 Two-Tier Monitoring Strategy

To improve translational feasibility, S-state monitoring should be organized as a two-tier dashboard rather than a platform dependent only on high-cost spatial technologies [163]. Tier 1 uses routine-accessible pathology-based methods, including H&E, IHC, multiplex IF, and digital pathology [169]. This level can support first-pass screening of dominant metabolic–immune axes. For example, LDHA/MCT4/CAIX combined with CD8 spatial exclusion may suggest an S1-like lactate–acidity barrier; CPT1A/PGC-1α/TOMM20 or OXPHOS markers may suggest S2-like persistence; G6PD/PHGDH/MTHFD2/xCT/GPX4 may indicate S3-like reductive defense; ACLY/ACSS2/FASN/SREBP1/ACSL4 may suggest S4-like lipid remodeling; and ACSL4/GPX4/xCT/4-HNE/MDA may help infer S5-related ferroptosis vulnerability or defense.

Tier 2 uses advanced spatial and metabolic platforms, including MSI, ST, single-cell omics, metabolic imaging, OCR/ETC assessment, lactate/pH mapping, lipid profiling, and redox-related readouts [166]. These approaches refine mechanistic resolution, distinguish tumor-cell versus stromal or immune-cell sources, and validate whether a Tier 1 pattern truly reflects a dominant metabolic barrier [153]. Thus, Tier 1 is intended for screening and prioritization, whereas Tier 2 is intended for validation, mechanistic interpretation, and trial-level stratification.

### 7.4 Minimal Feature Set for Calling S1–S5 and Intervention Considerations

To make the S1–S5 framework more operational, we propose a minimal feature set for inferring dominant metabolic-state tendencies in TNBC samples and tumor regions. This panel is not intended to define fixed molecular subtypes or absolute diagnostic categories. Instead, it is designed to support three practical goals: identifying the dominant metabolic barrier at a given time point, guiding intervention priorities across Phase I–III strategies, and tracking state switching or compensatory bypass during longitudinal treatment monitoring. Representative markers, functional readouts, spatial or immune features, and intervention priorities are summarized in Table 3.

**Table 3:** Minimal feature set for calling S1–S5 and intervention considerations.

State	Minimal Markers	Functional Readouts	Spatial/Immune Features	Intervention Priorities
S1 Glycolysis/lactate barrier (lactate–acidosis barrier)	GLUT1/HK2/PFKFB3 [9,13,14], LDHA, MCT4(±CAIX) [17,19,20]	Lactate enrichment/acidification tendency (e.g., MSI, metabolite- or tissue pH–related readouts; or ECAR-based evidence) [18,21]	A lactate–acidic “ring” is associated with reduced CD8^+^ infiltration and compromised effector function	Phase I: prioritize “wall-breaking” (block lactate production/export or neutralize acidity), then proceed to immune or cytotoxic attack [23,24,26,27,28]
S2 FAO/OXPHOS persistence barrier (mitochondrial persistence barrier)	CPT1A (or CPT1-related axis), PGC-1α/mitochondrial biogenesis signatures [34,35,37]	OXPHOS dependence and mitochondrial compensation (OCR/complex-activity evidence) [48,157]	Enrichment in post-therapy residual lesions/slow-cycling cells, indicating relapse risk [26,39,50,160]	Phase III: block glycolysis → OXPHOS bypass; Phase II: stagger with chemotherapy/ICB for synergy [39,50,160]
S3 NADPH/one-carbon defense barrier (NADPH–1C defense barrier)	G6PD/PPP axis; PHGDH–SHMT–MTHFD (one-carbon/serine pathway) [63,68]	NADPH/GSH buffering capacity and nucleotide demand (stress tolerance to radio/chemotherapy and replication stress) [75,77]	Stress-enriched regions are less sensitive to ROS-centric therapies and prone to DTP-like features [53,75,77]	Exploit “stress windows” with short-course barrier disruption; avoid prolonged single-node inhibition that provokes stronger compensation [81,117,120]
S4 Lipid enrichment/membrane remodeling barrier (lipid remodeling barrier)	ACLY/ACSS2 (acetyl-CoA supply), FASN, SCD1 (±SREBP axis) [92,93,97,102,103]	Lipid droplet/cholesteryl ester enrichment and membrane-lipid remodeling trend [91,94,112,113,114]	Invasive front/lipid-rich niches often co-segregate with immunosuppressive phenotypes [89,102,106,112]	Phase I/III: “dismantle lipid barriers + block nutrient provisioning”; stagger with ICB to enhance access/penetration [112,113,114,116]
S5 Ferroptosis shield/fate tipping point (ferroptosis shield/tipping point)	xCT (SLC7A)/GPX4 [72,120,123] (±FSP1/CoQ–DHODH axis), lipid-peroxidation signatures [124,125,130]	lipid-OOH accumulation vs. GSH/GPX4 buffering capacity [117,119,125]	A transient “vulnerability increase” window after stress/radiotherapy	Window-based attack: relieve defenses near the ROS/stress peak to trigger ferroptosis and amplify immune effects [117,120,125,128]

As summarized in Table 3, the value of this minimal feature set lies not in assigning a tumor to one fixed metabolic subtype, but in identifying dominant and targetable metabolic barriers. Therefore, longitudinal changes in these readouts may be more informative than isolated baseline values, especially for detecting therapy-induced state switching and compensatory resistance routes.

### 7.5 From Spatial Readouts to Dynamic Decision-Making

The main value of spatial monitoring is not to produce a static label, but to support dynamic decision-making [163]. First, spatial co-localization allows dominant barriers to be placed within specific tumor territories, such as acidic rings, lipid-rich invasive fronts, or perinecrotic stress zones [167]. Second, longitudinal trends are more informative than isolated absolute values, because sampling, platform, and normalization differences can limit cross-cohort comparability [163]. Third, axis-wise readouts can be linked to trial design by informing intervention timing, stop criteria, combination ordering, and maintenance-stage bypass blockade [163,164].

Machine-learning models, including ISMRS-like approaches, may help integrate clinicopathology, MSI, ST, IHC/IF, and imaging features into response or risk predictions [171,172]. However, these tools should remain interpretable and biologically anchored [171]. Their outputs should be mapped back to dominant barriers, state transitions, and therapeutic windows rather than used as black-box clinical classifiers [163]. Overall, spatial ecology provides the operational layer of the S1–S5 framework: it links metabolic state biology to tissue architecture, immune accessibility, therapy-induced remodeling, and biomarker-guided intervention hypotheses [4,7].

## 8 Therapeutic Strategy and Timing

**Figure 6:**
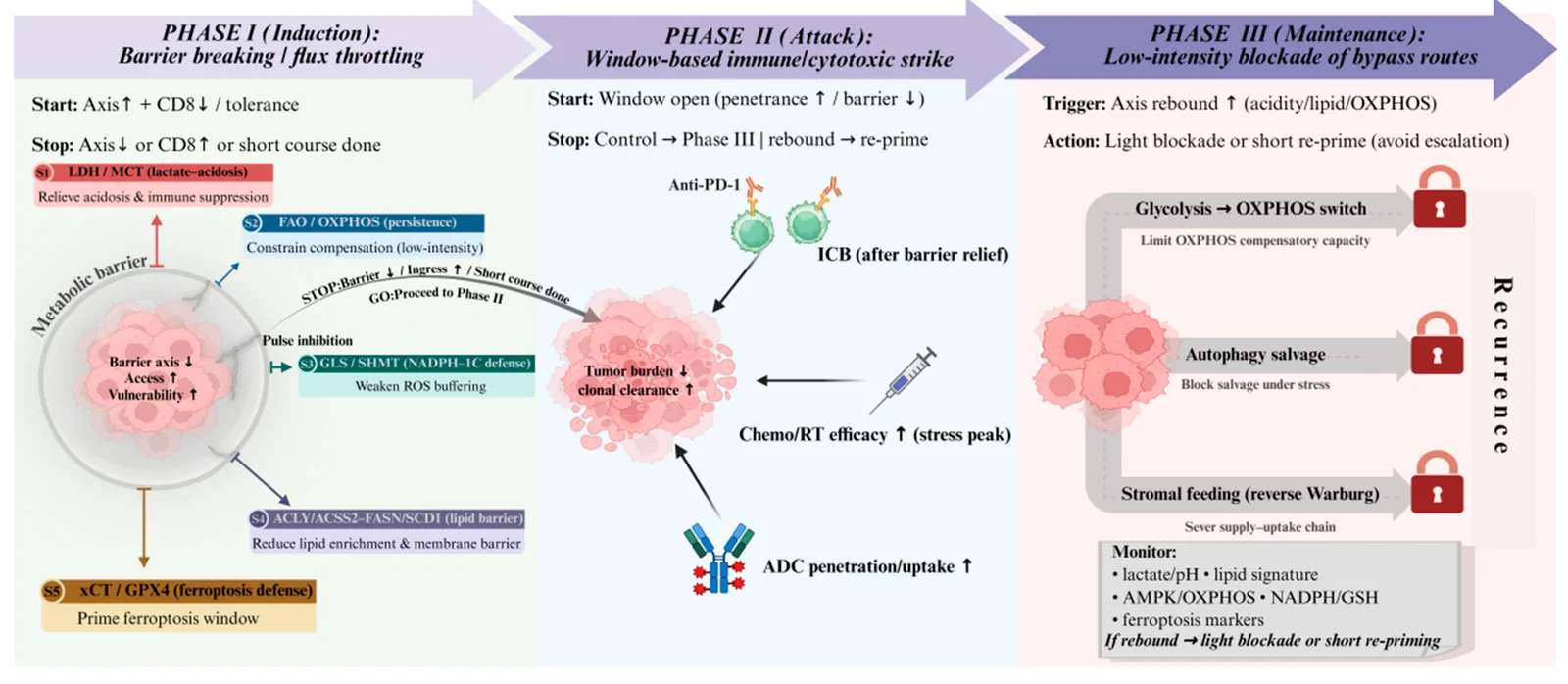
**Therapeutic strategy and timing: Phase I-III staged intervention logic**. Proposed temporal intervention logic that aligns metabolic plasticity with treatment timing. Phase I (Induction) uses short, pulsed barrier breaking/flux throttling guided by dominant axis signals (e.g., lactate–acidosis, OXPHOS persistence, NADPH/one-carbon defense, lipid enrichment, or ferroptosis defense) and prespecified start/stop criteria. Phase II (Attack) delivers window-based immune/cytotoxic strike (ICB, chemo/RT, ADCs) after barrier relief to maximize penetrance and killing. Phase III (Maintenance) applies low-intensity blockade of bypass routes (e.g., glycolysis → OXPHOS switching, autophagy salvage, stromal feeding) with monitoring-driven light re-priming to prevent recurrence. Created in BioRender.com.

The central challenge in TNBC is not whether a given pathway is upregulated, but whether the tumor can rapidly reprioritize metabolic flux under therapeutic pressure through “flux bypass”, while building spatial metabolic barriers that reinforce immune exclusion. Single-node, long-term metabolic suppression often provokes stronger compensation or inadvertently compromises immune effector cells [173]. Building on the S1-S5 state spectrum and the concept of spatial isozones, we propose a more translatable temporal strategy: Phase I barrier breaking/flux throttling (short course) → Phase II synergistic attack (immune/cytotoxic) → Phase III maintenance blockade of bypass routes (low intensity, monitorable, adjustable) [164]. Critically, each phase should be accompanied by observable “start/stop criteria”, and typical failure modes should have pre-specified rescue paths (Fig. 6).

### 8.1 Phase I: Short-Course Barrier Breaking and Flux Throttling

The goal of Phase I is to transiently weaken the dominant metabolic barrier and create a therapeutic window [174]. This phase should be short, pulsed, and reversible, because prolonged metabolic suppression may provoke stronger compensation or damage metabolically active immune cells [174]. Phase I should be initiated when monitoring indicates a dominant barrier axis, such as lactate–acidosis, lipid enrichment, OXPHOS-supported persistence, NADPH-centered defense, or ferroptosis-defense dominance, especially when these features are accompanied by restricted immune infiltration or signs of therapeutic tolerance [158].

For S1-dominant tumors, short-course targeting of lactate production, lactate export, or extracellular acidity may help relieve the lactate–acidic barrier and improve immune access [24,26,27]. Candidate strategies include targeting LDH, MCTs, CAIX, or acidity-buffering mechanisms [24,26,175], but these should be used as barrier-modifying approaches rather than indefinite glycolysis suppression. For S4-dominant tumors, transient inhibition of acetyl-CoA supply, fatty-acid synthesis, or membrane remodeling, such as ACLY/ACSS2/FASN/SCD1-related axes [102,103,112], may reduce lipid enrichment and improve access for immune effectors or antibody-based agents [112]. For S2-dominant or post-treatment residual tumors, Phase I should avoid further selecting mitochondrial bypass; instead, it should constrain FAO/OXPHOS compensation and prepare residual cells for Phase II attack [39,40,140]. For S3-dominant tumors, short-course weakening of NADPH, glutathione, or one-carbon defense may create a breach window for chemotherapy- or radiotherapy-induced oxidative stress [53,80,176]. If the aim is to exploit an S5 window, Phase I should focus on lowering ferroptosis defenses before or during the planned oxidative window, rather than sustaining hard pressure [117,125,177].

The stopping point of Phase I should be defined by barrier decline, improved immune ingress, completion of a preset short course, or emergence of toxicity risk [174]. Once the target barrier is sufficiently weakened, treatment should move rapidly into Phase II rather than extending metabolic inhibition until strong compensatory rewiring occurs [156].

### 8.2 Phase II: Immune or Cytotoxic Attack within the Opened Window

The goal of Phase II is true tumor-burden reduction [158]. After Phase I has weakened the dominant barrier, ICB, chemotherapy, radiotherapy, or ADCs should be delivered during the period of increased vulnerability. The key principle is temporal complementarity rather than simple drug stacking [164]. The attack modality should match the dominant state and the type of window created by Phase I. For S1-dominant tumors, relieving lactate–acidosis may increase CD8^+^ T-cell access and reduce immunosuppressive metabolic cues, thereby improving the rationale for ICB-based attack [18,21,27]. For S2-enriched residual disease, cytotoxic treatment may need to be paired with limited suppression of FAO/OXPHOS compensation to prevent DTP survival [32,39,40]. For S3-enriched tumors, radiotherapy or selected chemotherapies may be most effective when combined with short-window weakening of NADPH/GSH defense [53,176]. For S4-dominant tumors, lipid-barrier relief may improve ICB or ADC efficacy by reducing lipid-mediated immunosuppression, membrane remodeling, or drug-delivery resistance [91,112,113]. For S5-prone tumors, ferroptosis induction may be most rational during a transient oxidative window, potentially reinforcing immune activation through damage-associated molecular patterns and lipid-peroxidation-associated inflammatory signals [117,126]. Phase II should end when the intended tumor-control endpoint is reached or when monitoring indicates rebound compensation [164]. If metabolic compensation appears during or after attack, the strategy should shift toward Phase III maintenance rather than escalating the same intervention indefinitely.

### 8.3 Phase III: Maintenance Blockade of Bypass Routes

The goal of Phase III is to prevent residual clones from rebuilding growth capacity through compensatory bypass [158]. Unlike Phase II, this phase should prioritize low intensity, long-term tolerability, and monitoring-based adjustment [164]. Three bypass routes are especially relevant. First, glycolysis-to-OXPHOS switching can occur after prolonged pressure on S1-associated lactate pathways. In this setting, residual cells may activate AMPK-associated mitochondrial programs and increase dependence on fatty-acid or glutamine-fueled respiration [39,40,141]. Maintenance should therefore restrict mitochondrial compensation rather than further intensifying glycolysis inhibition [30,178]. Second, autophagy rescue can supply recycled amino acids, nucleotides, and lipids under metabolic stress; blocking this salvage route may reduce survival of residual cells [161,179]. Third, stromal feeding or reverse-Warburg-like support may allow CAFs or other stromal cells to provide lactate, pyruvate, lipids, or other substrates to tumor cells, thereby regenerating metabolic barriers and supporting relapse [154,155]. Phase III should be guided by longitudinal trajectories of monitoring axes. If acidosis resurges, lipid enrichment returns, OXPHOS compensation strengthens, or redox defense rebounds, short re-priming or lightweight blockade of the corresponding axis may be preferable to continuous high-intensity suppression [163,174]. This approach treats TNBC as an evolving metabolic system rather than a static target [163].

### 8.4 Translational Constraints and Safety Considerations

The proposed staged strategy must be interpreted within important safety and evidence boundaries. First, metabolic targets are rarely tumor-exclusive. Strong inhibition of OXPHOS, FAO, NADPH regeneration, one-carbon metabolism, or redox-buffering systems may cause cardiac, neurologic, gastrointestinal, hematologic, hepatic, or immune-related toxicity [49,50,87]. Therefore, short pulses, biomarker selection, and window-based intervention are more rational than indefinite systemic suppression [174]. Second, metabolic therapy may harm antitumor immunity if timing is inappropriate [145]. Activated effector T cells share some metabolic requirements with tumor cells, including glucose use, amino-acid availability, mitochondrial fitness, and redox control [18,145]. Thus, the sequence “break the barrier first, then immune attack” is intended to reduce tumor-imposed metabolic exclusion while avoiding prolonged suppression of immune-cell function. Third, spatial heterogeneity means that the same drug may have different effects in different tumor regions. A glycolytic intervention may weaken an acidic core but select OXPHOS-dependent residual cells elsewhere; lipid targeting may reduce invasive-front barriers but trigger compensatory nutrient uptake in other niches. Spatial co-registration and longitudinal sampling are therefore essential to avoid overinterpreting single-biopsy readouts [165,172].

Evidence level and reproducibility: most current evidence remains preclinical or correlative, and optimal combinations, doses, and timing require validation in rigorously designed cohorts and early-phase clinical trials [158]. The role of a review is to propose testable hypotheses and actionable trial frameworks, not a one-size-fits-all prescription. Finally, most timing strategies remain supported by preclinical models, correlative analyses, or early translational evidence rather than prospective clinical validation. The framework proposed here should therefore be used to generate testable trial hypotheses, including start/stop rules, intervention ordering, and maintenance-stage monitoring, rather than as an immediately prescriptive treatment schedule [158,163,164].

### 8.5 Summary

Overall, TNBC therapy should be conceptualized as a dynamic process shaped by metabolic state switching, spatial barrier remodeling, and adaptive bypass [173]. The S1–S5 framework suggests that therapeutic efficacy depends not only on choosing a biologically relevant target, but also on selecting the right timing, sequence, and combination. In this model, Phase I opens a window by weakening the dominant barrier, Phase II delivers immune or cytotoxic attack during that window, and Phase III limits bypass-driven relapse through low-intensity maintenance and monitoring. As single-cell, spatial, metabolic-imaging, and longitudinal profiling technologies mature, treatment design may progressively move from static subtype-based selection toward dynamic intervention guided by observable state transitions [169].

## 9 Challenges, and Future Directions

Metabolic reprogramming in TNBC links tumor-cell plasticity, spatial ecology, immune remodeling, and therapeutic resistance. The S1–S5 framework provides a structured way to organize these processes, but its clinical translation still faces several conceptual, technical, and therapeutic barriers. Therefore, the framework should be interpreted as a hypothesis-generating tool for biomarker development, longitudinal monitoring, and rational trial design rather than as a ready-to-use clinical classification system.

### 9.1 Current Challenges and Technical Bottlenecks

First, druggability and selectivity remain major obstacles. Many metabolic targets in TNBC, including mitochondrial respiration, lipid metabolism, NADPH regeneration, one-carbon metabolism, and redox-buffering systems, are also required by normal tissues. Therefore, systemic inhibition may produce narrow therapeutic windows and clinically relevant toxicity, especially in high-energy organs such as the heart and brain [49,50,87]. Unlike tumors driven by highly selective mutant metabolic enzymes, TNBC usually depends on exaggerated but physiologic metabolic programs, making broad and durable tumor selectivity difficult to achieve.

Second, metabolic plasticity creates extensive bypass circuitry [173]. When one metabolic route is blocked, TNBC cells may activate compensatory nutrient-acquisition programs, including autophagy, macropinocytosis, mitochondrial adaptation, or alternative substrate use [128,161,173]. Some tumor cells may also maintain hybrid metabolic phenotypes with both glycolytic and oxidative capacity, allowing flexible survival under single-pathway inhibition [4,173]. This explains why long-term single-node metabolic suppression often fails and why combination timing may be as important as target selection.

Third, spatial metabolic monitoring remains technically challenging. Spatial metabolomics and spatial transcriptomics can reveal tissue-level metabolic heterogeneity, but current platforms remain expensive, relatively low-throughput, and insufficiently standardized for routine clinical use [165,166]. Single-cell metabolite detection also faces limitations in sensitivity, coverage, and reproducibility. Moreover, spatial transcriptomics can infer metabolic programs from enzyme-expression patterns, but it cannot fully replace direct measurements of metabolites or flux. Thus, clinical translation requires simplified, reproducible, and tiered readout systems rather than reliance on a single advanced platform.

Fourth, dynamic state switching is difficult to capture using conventional biopsies [163]. A single pretreatment sample may miss therapy-induced transitions, such as S1-to-S2 mitochondrial compensation or S3-mediated redox defense activation [53,178]. Therefore, future clinical workflows should prioritize longitudinal and axis-wise monitoring, using changes in lactate–acidity, FAO/OXPHOS persistence, NADPH defense, lipid remodeling, and ferroptosis vulnerability to guide treatment adjustment [163].

Fifth, metabolic intervention may have double-edged effects on antitumor immunity [145]. Activated CD8^+^ T cells share several metabolic requirements with tumor cells, including glucose utilization, amino-acid uptake, mitochondrial fitness, and redox balance [21,145]. Therefore, indiscriminate metabolic blockade may weaken immune effector function even while suppressing tumor metabolism. A key unresolved challenge is how to remodel tumor-protective metabolic barriers without impairing immune-cell fitness [145,176].

### 9.2 Future Directions and Technological Frontiers

Future studies should first improve spatial and single-cell metabolic profiling [166]. Emerging single-cell and spatial profiling approaches may help resolve metabolic heterogeneity and spatially organized metabolic programs at higher resolution [153,166]. These approaches could help localize rare but clinically important subpopulations, such as dormant S2-like residual cells, S3-like stress-resistant cells, or S5-prone ferroptosis-vulnerable regions [158].

Second, AI-driven metabolic network modeling may help integrate multi-omics, spatial, pathological, and clinical information into testable prediction models [180]. Such models could infer dominant metabolic states, simulate responses to metabolic perturbation, and prioritize drug combinations or treatment sequences [180]. Early computational frameworks, such as deep-learning-based drug-combination prediction [181], support the feasibility of this direction. However, these models should remain interpretable and biologically anchored, with outputs mapped back to dominant barriers, flux-valve nodes, and therapeutic windows rather than used as black-box recommendations [180].

Third, next-generation tumor-selective metabolic inhibitors are needed. Prodrug strategies, tumor-activated compounds, and isozyme-selective allosteric inhibitors may widen therapeutic windows [50,88,182]. For example, the glutamine antagonist JHU has been designed to improve tumor-selective activation and reduce systemic toxicity, while also potentially reshaping antitumor immunity [141,183]. Similar principles may be applied to OXPHOS, one-carbon metabolism, lipid remodeling, or redox-defense pathways, especially when paired with biomarker-defined windows [176,182].

Finally, precision metabolic therapy should incorporate interactions among tumor cells, immune cells, stromal cells, and the microbiome. Recent evidence suggests that intratumoral bacteria may influence chemotherapy response by altering drug availability and substrate metabolism [184]. Therefore, future TNBC strategies may need to integrate host metabolism, immune ecology, stromal remodeling, diet, and microbiome-related interventions into a broader metabolic precision-medicine framework [18,155].

Overall, the next stage of TNBC metabolic research should move from pathway description toward dynamic and spatially resolved intervention design. The key questions are no longer simply which metabolic pathway is active, but when it becomes dominant, where it forms a barrier, whether it creates a therapeutic window, and how it should be targeted without triggering compensatory escape or immune impairment.

## 10 Conclusion

TNBC metabolic plasticity should not be viewed simply as an unmanageable collection of pathway alterations. Across microenvironmental and therapeutic contexts, available evidence suggests that TNBC cells repeatedly converge on several functional metabolic priorities, including glycolysis–lactate-driven barrier formation, FAO/OXPHOS-supported persister-like survival, NADPH/one-carbon-mediated reductive defense, lipid remodeling, and ferroptosis-related vulnerability. The S1–S5 framework organizes these recurrent programs into a dynamic operating map that links metabolic flux redistribution to spatial heterogeneity, immune suppression, drug tolerance, and state-specific therapeutic windows.

This framework is not intended to replace established molecular classifications or serve as a validated clinical treatment algorithm. Instead, its practical value lies in reframing TNBC metabolism around a more actionable question: whether a dominant operating state or transition route can be detected, monitored, and exploited at a specific therapeutic time point. Thus, S1–S5 should be interpreted as functional, reversible, and context-dependent operating states rather than fixed tumor subtypes.

Future translation will require practical state readouts, biomarker-guided intervention windows, and prospective validation. Routine-accessible tools such as H&E morphology, immunohistochemistry, multiplex immunofluorescence, and digital pathology may support first-tier screening, whereas spatial omics, metabolic imaging, and longitudinal profiling can refine mechanistic resolution. Overall, the S1–S5 framework provides a structured and falsifiable hypothesis generator for understanding TNBC metabolic adaptation and designing future biomarker-driven therapeutic strategies.

## Data Availability

Not applicable.

## Abbreviations

ACADs	Acyl-CoA dehydrogenases
ACAT1	Acyl-CoA:cholesterol acyltransferase 1
ACC	Acetyl-CoA carboxylase
ACLY	ATP-citrate lyase
ACSL4	Acyl-CoA synthetase long-chain family member 4
ACSS2	Acyl-CoA synthetase short-chain family member 2
ADC	Antibody-drug conjugate
AhR	Aryl hydrocarbon receptor
AI	Artificial intelligence
AMPK	AMP-activated protein kinase
ARG1	Arginase 1
ATP	Adenosine triphosphate
BCL-XL	B-cell lymphoma-extra large
BL1	Basal-like 1
BL2	Basal-like 2
BLIA	Basal-like immune-activated
BLIS	Basal-like immune-suppressed
BrM	Brain metastasis
BRQ	Brequinar
CAF	Cancer-associated fibroblast
CAFs	Cancer-associated fibroblasts
CAIX	Carbonic anhydrase IX
CD36	Cluster of differentiation 36
CD47	Cluster of differentiation 47
CoQ10	Coenzyme Q10
COX-2	Cyclooxygenase 2
CPT1	Carnitine palmitoyltransferase 1
CPT1A	Carnitine palmitoyltransferase 1A
CSC	Cancer stem cell
DESI-MSI	Desorption electrospray ionization mass spectrometry imaging
DHODH	Dihydroorotate dehydrogenase
DMOCPTL	Dimethylaminomicheliolide-like parthenolide derivative
DTP	Drug-tolerant persister
DTPs	Drug-tolerant persisters
ECAR	Extracellular acidification rate
ECM	Extracellular matrix
EGFR	Epidermal growth factor receptor
EMT	Epithelial-to-mesenchymal transition
ETC	Electron transport chain
F-2,6-BP	Fructose-2,6-bisphosphate
FAO	Fatty acid oxidation
FASN	Fatty acid synthase
FSP1	Ferroptosis suppressor protein 1
G6PD	Glucose-6-phosphate dehydrogenase
GLS	Glutaminase
GLUD	Glutamate dehydrogenase
GLUT1	Glucose transporter 1
GPX4	Glutathione peroxidase 4
GSH	Glutathione
H&E	Hematoxylin and eosin
HER2	Human epidermal growth factor receptor 2
HIF-1α	Hypoxia-inducible factor-1α
HK2	Hexokinase 2
ICB	Immune checkpoint blockade
IDH1	Isocitrate dehydrogenase 1
IDH2	Isocitrate dehydrogenase 2
IDO1	Indoleamine 2,3-dioxygenase 1
IF	Immunofluorescence
IFN-γ	Interferon gamma
IHC	Immunohistochemistry
ISMRS	Isozone state and metabolic response score
Kyn	Kynurenine
LAR	Luminal androgen receptor
LDH	Lactate dehydrogenase
LDHA	Lactate dehydrogenase A
lipid-OOH	Lipid hydroperoxide
LXR	Liver X receptor
MALDI-MSI	Matrix-assisted laser desorption/ionization mass spectrometry imaging
MCT	Monocarboxylate transporter
MCT1	Monocarboxylate transporter 1
MCT4	Monocarboxylate transporter 4
MDA	Malondialdehyde
MDSC	Myeloid-derived suppressor cell
MDSCs	Myeloid-derived suppressor cells
ME1	Malic enzyme 1
ME2	Malic enzyme 2
MHC	Major histocompatibility complex
MTHFD	Methylenetetrahydrofolate dehydrogenase/cyclohydrolase
mTOR	Mechanistic target of rapamycin
mTORC1	Mechanistic target of rapamycin complex 1
MUFA	Monounsaturated fatty acid
MYC	MYC proto-oncogene
NAD^+^	Nicotinamide adenine dinucleotide
NADH	Reduced nicotinamide adenine dinucleotide
NADPH	Nicotinamide adenine dinucleotide phosphate
NAMPT	Nicotinamide phosphoribosyltransferase
NCOA4	Nuclear receptor coactivator 4
NDUFA4L2	NADH:ubiquinone oxidoreductase subunit A4-like 2
NF-κB	Nuclear factor kappa B
NFAT	Nuclear factor of activated T cells
NRF2	Nuclear factor erythroid 2-related factor 2
OCR	Oxygen consumption rate
OS	Overall survival
OXPHOS	Oxidative phosphorylation
PARP	Poly(ADP-ribose) polymerase
PARPi	Poly(ADP-ribose) polymerase inhibitor
PC	Pyruvate carboxylase
pCR	Pathological complete response
PD-1	Programmed cell death protein 1
PD-L1	Programmed death-ligand 1
PDH	Pyruvate dehydrogenase
PDK1	Pyruvate dehydrogenase kinase 1
PFKFB3	6-phosphofructo-2-kinase/fructose-2,6-bisphosphatase 3
PFS	Progression-free survival
PGC-1α	Peroxisome proliferator-activated receptor gamma coactivator 1-alpha
PGE2	Prostaglandin E2
PGRMC1	Progesterone receptor membrane component 1
PHGDH	Phosphoglycerate dehydrogenase
PKM2	Pyruvate kinase M2
PPP	Pentose phosphate pathway
Prx3	Peroxiredoxin 3
PTEN	Phosphatase and tensin homolog
PUFA	Polyunsaturated fatty acid
RARRES2	Retinoic acid receptor responder 2
ROS	Reactive oxygen species
SCD1	Stearoyl-CoA desaturase 1
SGOC	Serine-glycine-one-carbon
SHMT	Serine hydroxymethyltransferase
SIRT3	Sirtuin 3
SLC7A11	Solute carrier family 7 member 11
SOAT1	Sterol O-acyltransferase 1
SOCS1	Suppressor of cytokine signaling 1
SOD2	Superoxide dismutase 2
SREBP	Sterol regulatory element-binding protein
SREBP1	Sterol regulatory element-binding protein 1
SREBP2	Sterol regulatory element-binding protein 2
ST	Spatial transcriptomics
TAM	Tumor-associated macrophage
TAMs	Tumor-associated macrophages
TCA	Tricarboxylic acid
Teff	Effector T cell
TGF-β	Transforming growth factor beta
TME	Tumor microenvironment
TNBC	Triple-negative breast cancer
TOMM20	Translocase of outer mitochondrial membrane 20
Treg	Regulatory T cell
Tregs	Regulatory T cells
xCT	Cystine/glutamate antiporter
4-HNE	4-hydroxynonenal
